# Dogs of War: The Effect of War‐Inflicted Environmental Damage on Free‐Ranging Domestic Dogs

**DOI:** 10.1111/eva.70182

**Published:** 2025-12-05

**Authors:** Mariia Martsiv, Ihor Dykyy, Małgorzata Witek, Piotr Chibowski, Giulia Cimarelli, Andre E. Moura, Małgorzata Pilot

**Affiliations:** ^1^ Ivan Franko National University of Lviv Lviv Ukraine; ^2^ Faculty of Biology University of Gdańsk Gdańsk Poland; ^3^ Faculty of Biology, Biological and Chemical Research Centre University of Warsaw Warszawa Poland; ^4^ Domestication Lab, Konrad Lorenz Institute of Ethology University of Veterinary Medicine Vienna Vienna Austria; ^5^ Behavioural Ecology Group Wageningen University and Research Wageningen the Netherlands

**Keywords:** anthropogenic disaster, contemporary evolution, domestic dogs, mortality, natural selection, war‐inflicted environmental damage

## Abstract

Wars impose unprecedented environmental damage that has rarely been studied in real time. Domestic dogs are an accessible model species during war times, because they enable data collection without specialised equipment and skills, which can be performed without creating additional danger to humans or animals involved. We compared phenotypic traits in Ukrainian dogs living close to the front line with those from other regions of Ukraine. We found significant differences in frequencies and diversity of multiple morphological traits, consistent with mortality‐based selection at the front line. We also found differences in age structure and frequency of diseases and injuries, consistent with high mortality of old and ill individuals. The front‐line population had low average BMI and stable isotope analysis suggested malnutrition and low trophic level. Our study shows that wars can be factors of strong and fast natural selection, with the effects comparable to large‐scale natural or anthropogenic disasters.

## 1 Introduction

War has been pervasive throughout human history. With the inventions of increasingly powerful weapons, the impact of wars has intensified in both their destructive power and geographic range, affecting entire ecosystems. This has culminated in the 20th century, when wars achieved global reach, leading altogether to over 100 million human deaths (Leitenberg 2006), with animal deaths unaccounted for. Among anthropogenic causes of environmental change, modern wars are most intense and have immediate and far‐reaching consequences (Machlis and Hanson 2008; Lawrence et al. 2015). Although most wars have a geographically contained impact, they strongly contribute to global environmental change (Vogler 2024). It has been estimated that military training alone, without active wars, accounts for about 10% of global carbon emissions annually and uses between 750,000 and 1,500,000 km^2^ of land (Biswas 2000; Majeed 2004; Machlis and Hanson 2008). Wars are therefore in direct opposition to humanity's strive towards a sustainable future (Editorial 2023).

Knowledge on the environmental impacts of military activities is relatively scarce, and most studies focused on this topic explore the environmental conditions only after the end of military activities and without data on baseline conditions (Lawrence et al. 2015). Some knowledge of this topic comes from studies carried out in military training sites. For example, in mule deer ( *Odocoileus hemionus* ), significant differences in home range use were observed between areas used for military training and control areas. Observed differences included temporarily abandoning the training areas as well as increasing movement distances during the periods of training activity (Stephenson et al. 1996). In Blainville's beaked whales ( *Mesoplodon densirostris* ), military exercises using active sonars resulted in an avoidance of the affected area (Jones‐Todd et al. 2022). In contrast, black bear ( *Ursus americanus* ) showed no change in habitat use in response to noise generated from weapons‐firing exercises, except for avoidance of small areas near the firing positions (Telesco and Van Manen 2006). Similarly, reproductive success in red‐cockaded woodpecker ( *Picoides borealis* ) was not significantly affected by infrequent, short‐duration military exercises (Delaney et al. 2011). However, the frequency, duration, intensity and spatial scale of military exercises do not reflect the actual military confrontations. Although studies on wildlife at military training sites bring important knowledge on the type of impacts that can be expected from armed conflicts, they do not fully reflect the environmental pressures experienced during prolonged military conflicts. Moreover, the effect of wars is not limited to military confrontations but also includes other long‐lasting environmental changes, for example, human density declines and the collapse of agriculture.

Environmental damage caused by wars has rarely been studied in real time, given the multitude of more urgent problems experienced by affected human populations and challenges associated with carrying out research in active conflict zones. However, war‐inflicted environmental damage may have very significant immediate consequences in both natural and human‐modified habitats, resulting in shifts in the structure of both natural and artificial food chains that affect human populations as well as wild and domestic animals (Dudley et al. 2002; Gaynor et al. 2016; Daskin and Pringle 2022; Weir 2023). Areas subject to the strongest direct damage are human‐dominated landscapes that are affected by intentional destruction of infrastructure and areas on the front line that are damaged as a result of military activities. Due to the very fast rate of these environmental changes, the full scope of their consequences may be difficult to assess retrospectively.

The real‐time assessment of the effects of wars on animals and ecosystems may be achieved by studying migratory species using remote‐sensing techniques such as satellite telemetry (Russell et al. 2024), but such species have the greatest capabilities to avoid the most dangerous regions. Therefore, the most likely effect they experience is the modification of migratory routes and associated fitness consequences. Non‐migratory species experience the strongest consequences of military activities both at the individual (changes in behaviour, physiology and diet) and population levels (demographic changes, natural selection). Therefore, studying such species may provide better insight into the ecological impacts of wars. However, field studies on wild animals require the on‐site presence of qualified staff, which is impossible in active war zones. An alternative approach may be to focus on organisms that are relatively easy to study. Domestic dogs may be considered an accessible model species during wartime because the data collection from them does not require the specialised skills, equipment and logistic effort necessary to study wildlife. Therefore, data collection can be performed in a simpler way and without creating additional danger to the humans involved or the dogs. Dogs are popular companion animals and therefore their life history (in both the ecological and colloquial sense of the phrase) is linked to that of the humans they are associated with, during both peace and wartime (Beck 2013).

Dogs inhabit human‐dominated landscapes and therefore are directly affected by wars. Some of the direct effects they experience are likely to be similar to those experienced by humans: drastic increases in mortality rates, changes in interaction patterns with conspecifics and with humans, as well as restrictions and changes in food supplies. Owned dogs may become translocated together with their owners, may change owners or lose owners altogether and become stray. Chained dogs may gain freedom, newly stray dogs may form new social bonds with conspecifics, and group sizes and social interactions of free‐ranging dogs may change. Limitations in direct and indirect (e.g., via dumps) food provision by humans may lead to a shift towards alternative food sources, for example, hunting domestic animals and wildlife, or scavenging (Ritchie et al. 2014). Survival in such altered circumstances may be non‐random and related to specific phenotypic characteristics, involving both heritable and plastic traits such as body mass index (BMI). War time is thus likely to be associated with strong natural selection, but it is unknown which heritable phenotypic traits are affected and which plastic traits have the strongest effect on survival.

The majority of dogs globally can breed freely and do not belong to any particular breed (Hughes and Macdonald 2013). This is also the case in Ukraine, where such dogs are found among owned pets in villages and cities, as well as among stray dogs. Free‐breeding dog populations show moderate genetic differentiation across Europe (Pilot et al. 2015) and therefore limited differentiation may be expected within Ukraine before the war. Dogs that can range freely for at least part of their time and are unrestricted in their mate choice, are subject to natural and sexual selection pressures similar to wild animal populations (Pilot et al. 2016; Liu et al. 2021), and therefore they were likely to be under strong selective pressures during the war.

Some phenotypic traits facilitating survival of dogs under extreme environmental pressures during wartime may be undesirable from the human perspective. For example, free‐ranging dogs may have the tendency to form large social groups, which may facilitate hunting and/or defending food sources when human‐derived food is scarce. Dogs occurring in groups pose a higher risk of attacks on humans and a higher likelihood of serious consequences for the victims (Borchelt et al. 1983; Raghavan 2008; Santoro et al. 2011). Large social groups also present a higher risk of spreading infectious diseases, including those dangerous to humans, livestock and wildlife, for example, rabies (Kartal and Rowan 2018). In addition, increased numbers of stray dogs combined with reduced access to human‐derived food may amplify the negative impact of dogs on wild animals that are targeted as prey (Young et al. 2011; Hughes and Macdonald 2013). Some problems are specifically related to wartime and have rarely been addressed. For example, it is unknown whether scavenging of dogs on dead human bodies, sometimes reported from war zones, is an extreme behaviour occurring only on rare occasions, or it may instead be considered a typical behaviour of scavengers, expected to occur whenever triggered by predictable circumstances (see Borchelt et al. 1983).

In this study, we provide data that shed light on the selective pressures on dog populations and the welfare of individual dogs during war times. This includes the analysis of morphological traits in individual dogs based on measurements and photographs, the analysis of body condition through assessment of visible diseases or injuries, the calculation of BMI, the inference of diet using stable isotope analysis, and the assessment of age structure. We analyse dogs from different regions of Ukraine that have been affected by the war to various extents. We predict that natural selection will act against phenotypes that may reduce fitness in free‐living dogs, such as short paws, floppy ears and deviations from the mesocephalic (i.e., regular) snout shape. Therefore, we expect reduced frequencies of these phenotypes and reduced variation in each trait (i.e., in snout and ear shape and paw length) in dogs living close to the front line. In addition, we assess the differences in group size, as group formation is an important behavioural strategy that facilitates hunting and resource defence. Identifying the factors affecting dog survival may help devise efficient strategies to improve the welfare of stray dogs in regions affected by wars and reduce the risk of dog attacks on humans and the spread of infectious diseases. This knowledge will also improve the general understanding of the impact of wars on animals and the environment.

## 2 Methods

### 2.1 Data Collection

**FIGURE 1:**
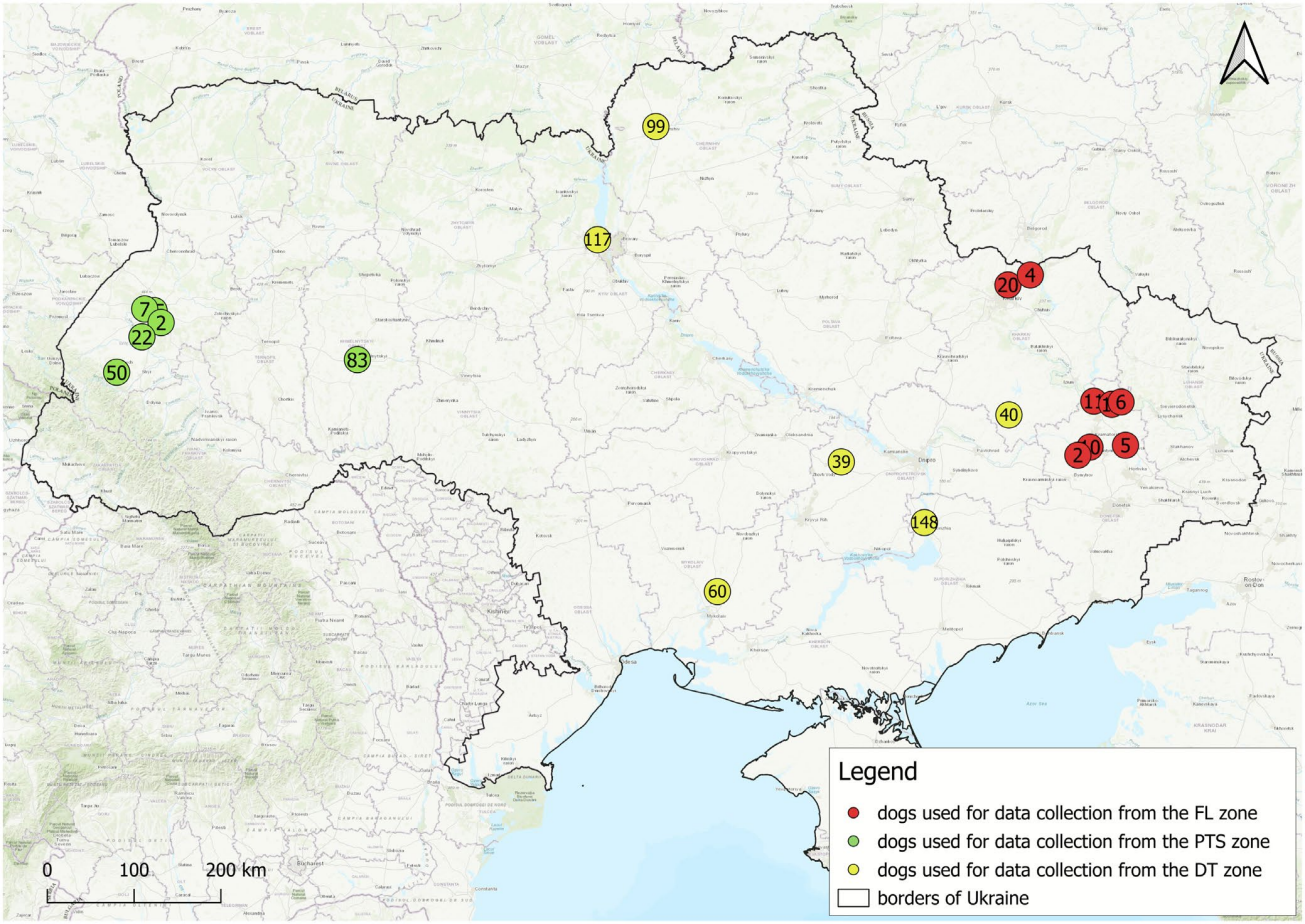
Distribution of dogs used for the collection of phenotypic data and hair samples in three parts of Ukraine categorised by the intensity of military activities: DT, dangerous territories (503 dogs); FL, front line (73 dogs); PST, potentially safe territories (219 dogs). Locations are clustered to clearly visualise the number of dogs in each area.

This study was designed in response to the war in Ukraine and carried out during the war. Therefore, the methods of data collection were limited to those that can be applied safely, rapidly and without the need for any specialised equipment. Between 17th of March 2023 and 10th of January 2024, we obtained measurements (weight, height, leg length), information about health status and age group, as well as photographs and hair samples from 763 dogs from 9 regions of Ukraine (*oblast* in Ukrainian; Figure 1).

The data and photographs were collected by staff of animal shelters, veterinarians and volunteers helping stray animals, who lived and worked in the places of sample collection. The data from dogs living at the front line were obtained by soldiers. No‐one travelled to dangerous regions for the purpose of this study. All data were obtained using the same protocol, using only simple instruments (weighting scale, meter, a mobile phone camera and a Google map used to establish geographic coordinates of data collection). We could not obtain all the data from all individuals assessed because of technical limitations (e.g., unavailability of a weighting scale); therefore the sample sizes differ between the traits assessed.

We collected data only from dogs that did not belong to any breed and were not a mix of two breeds. Of 763 dogs studied, 170 (22.3%) were stray, while the remaining individuals either had owners or lived in shelters. These two categories of dogs were not clearly separated; for example, some dogs could be free‐ranging but have an owner/carer who fed them, and some owned dogs were adopted from shelters. It should be stressed that aggressive and very shy stray dogs could not be sampled. For such individuals, we could only obtain photographs, as described below.

The data and sample collection were carried out in nine regions: Lviv (137), Kyiv (117), Chernihiv (99), Zaporizhia (148), Khmelnytskyi (83), Mykolaiv (60), Dnipro (39), Kharkiv (56), Donetsk (24) (Figure 1). Some of these dogs (211 individuals, 27.7%) were moved from other regions of Ukraine as a result of the war. In such cases, we considered as the dog's location the place where the dog currently lived. The largest number of relocated dogs (66) were from the Kherson Oblast. Most of them were displaced in June 2023, after the destruction of the Kakhovka Dam, an intentionally imposed environmental disaster. Dogs were also moved from the Kharkiv Oblast (38), Donetsk Oblast (14), Zaporizhia Oblast (14), Kyiv Oblast (13) and Chernihiv Oblast (12), and other regions (1–8 individuals).

**FIGURE 2:**
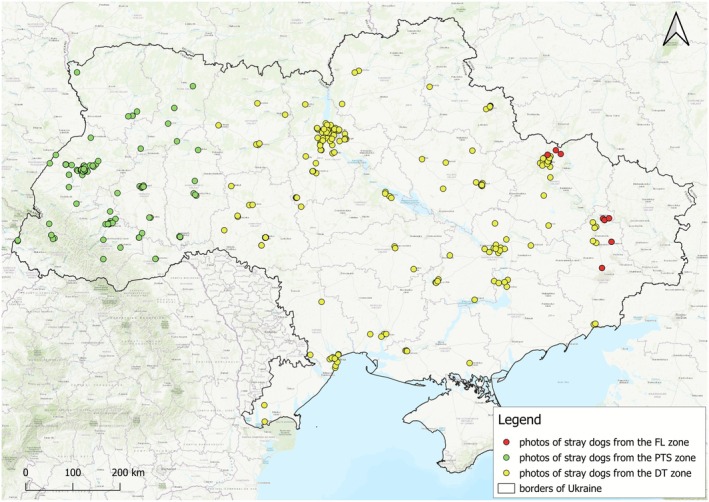
Distribution of photos of stray dogs taken in three parts of Ukraine categorised by the intensity of military activities: DT, dangerous territories (198 photos); FL, front line (35 photos); PST, potentially safe territories (139 photos). The front line was defined as the zone of direct military confrontation and the samples from this zone were collected by soldiers only. The spatial overlap between FL and DT results from temporal changes in the location of the front line.

Besides dogs that were directly approached to collect the measurement data and hair samples, we also collected photographs from stray dogs without approaching them in order to obtain information about the proportion of dogs occurring in social groups and the sizes of such groups. This approach is not unbiased, because social group members do not always occur together. Nevertheless, multiple individuals occurring together are indicative of groupings, so we were able to identify cases when individuals stayed in close associations, even if these associations did not represent entire social groups. We obtained 381 photographs from all regions of Ukraine except Luhansk Oblast and Crimea (23 regions, Figure 2), taken between 2nd of April 2023 and 16th of January 2024.

The stray dogs photographed were not approached for the data collection and photographs of 763 individuals that were used for the data collection, as described above, were not used in the assessment of the group size. However, in order to increase the sample size for the front line (FL) dogs, we used the photographs of stray dogs from FL to obtain the phenotypic data (e.g., snout shape, ear shape, coat colour). Therefore, the only overlap between the dataset used to obtain the phenotypic data and the dataset used to assess the group sizes was for the FL dogs.

The locations of the dog measurements, hair samples and photographs' collection were divided into three zones depending on how intensely they were affected by the war (Figures 1 and 2): (1) regions under the direct influence of war in parts of the Donetsk and Kharkiv regions (FL—front line; 76 dogs); (2) dangerous territories in central and eastern Ukraine where shelling occurs frequently (DT; 503 dogs); (3) potentially safe territories in western Ukraine (PST; 219 dogs). The front line (FL) was defined as the areas of direct military activities. The data and photographs in the FL were collected by soldiers only and therefore the FL is defined by the presence of soldiers. The spatial delimitation of these three zones is consistent with the interpolated map of the intensity of military activities, including the steep transition between DT and FL (Figure 5B).

The data collection was carried out for 10 months and the front line has been changing its location during that period. The FL locations included parts of Donetsk Oblast and parts of Kharkiv Oblast; the front line extent is longer but we were not able to obtain the data from sections not presented here. The second group, the dangerous territories (DT) included dogs from territories where shelling occurs often (Zhytomyr, Kyiv, Chernihiv, Vinnytsia, Kirovohrad, Cherkasy, Zaporizhzhya, Poltava, Sumy, Dnipro, Odesa, Kherson, Mykolaiv, as well as part of Kharkiv and Donetsk Oblasts), and the third group, the potentially safe territories (PST) included dogs from territories where shelling is less frequent (Zakarpatia, Chernivtsi, Ivano‐Frankivsk, Lviv, Volyn, Ternopil, Khmelnytskyi, and Rivne Oblasts). The division between DT and PST was based on the administrative borders of oblasts from western versus central and eastern Ukraine. The measurements and hair samples were only collected from two western regions of Ukraine (Lviv Oblast, Khmelnytskyi Oblast) distant from sampling locations in central Ukraine, so in this case the division was clear (Figure 1). The photographs were obtained from a broader geographic range compared with the dogs from which the measurements were taken, and the same division was maintained for the purpose of data analysis (Figure 2), even though the frequency of shelling does not change discretely according to the administrative borders.

### 2.2 Height, Weight, BMI and Leg Length

The height of individuals was obtained by taking a measure at the withers. Whenever possible, the weight of the dog was measured using a scale. To calculate the body mass index (BMI), the body height in cm was converted to inches and the body weight in kg was converted to lbs (pounds). BMI was calculated by dividing the body weight in lbs by the body height in inches. In addition, the length of a front leg was measured in order to distinguish short‐legged dogs from those with regular leg length. For this purpose, we calculated the proportion of leg length to body height. We analyzed this proportion as a continuous variable, but we also used it to create two categories of leg length. If leg length was less than 50% of the body height, legs were classified as short; otherwise they were classified as regular length.

We created categories for height, weight and BMI based on ranges of values and calculated frequencies of individuals within each category. Based on these frequencies, we calculated the Simpson's index D (Simpson 1949) and used 1‐D as a measure of diversity for each trait.

### 2.3 Characterisation of Morphological Traits

We assessed the following morphological traits of dogs based on their photographs:

– Snout shape: brachycephalic, mesocephalic, dolichocephalic.
– Ear shape: straight, half‐floppy, floppy, one floppy—one half floppy, one half floppy—one straight, intentionally cut.
– Tail shape: straight, half‐curly, curly.
– Hair type: straight, half‐curly, curly.
– Hair length: short, middle, long, very long.
– Coat colour patterns (see Table S1).

The coat colour patterns were assessed using a system designed for another study (Cimarelli and colleagues, unpublished; see Table S1) based on the known genetic background of coat coloration in dogs (Brancalion et al. 2022).

### 2.4 Age, Injuries and Diseases

We used photographs to assign dogs to age categories: young, adult, and old. Pups and subadults who clearly did not look as mature individuals were classified as young. Individuals who showed clear signs of aging, such as greying fur, were classified as old.

The dogs were examined for visible diseases or injuries, and the shelter dogs were additionally assessed by veterinarians, who provided diagnoses of their diseases. Because not all animals studied could be assessed by a veterinarian, we did not focus on precise diagnoses, but instead classified the diseases and injuries into broad categories: leg injuries or disabled/missing legs; eye diseases and eye loss; wounds and scars; skin diseases; and other diseases. This last category included body injuries, spine damage, facial injuries, deafness, glaucoma, *Demodex* mites, rhinitis, salivary gland tumour, kidney failure, pyometra, sarcoma, contusion, epilepsy, venereal sarcoma, tumours, arthritis, kidney stone disease, and alopecia. Among the reported health problems there was also considerably reduced weight or excessive weight, but the body mass and BMI were assessed separately and therefore these health problems were not considered with other diseases.

### 2.5 Stable Isotope Analysis

We analysed stable isotope composition of carbon and nitrogen in 97 hair samples of Ukrainian dogs (Table S2) to obtain information about their diet. The hair samples originated from five sampling sites: Lviv and Truskavets in Lviv Oblast (PST zone), Dnipro and Zaporizhia Oblasts (DT zone) and Donetsk Oblast (FL zone). Stable carbon and nitrogen isotopic composition measurements were carried out at the Laboratory of Biogeochemistry and Environmental Protection, Biological and Chemical Research Centre, University of Warsaw. Whenever possible, we selected guard hair for the analysis, but some samples included only very thin hair which resembled underfur. A hair sample consisted of 10–30 individual hairs, depending on their length and thickness. Each hair sample was divided into two subsamples representing different hair growth phases, which were measured separately. Average difference between the older and the most recent part was 0.072‰ for δ^13^C and −0.073‰ for δ^15^N, which was small compared with the estimated error rate (see below). The analysis of temporal changes in individual diet based on the differences between the values obtained from the two parts of the hair gave no clear conclusions (see Data S1). Therefore, we used mean values from the measurements of two parts of each hair sample for further data analysis.

Each sample (whisker part or bundle of hair halves) was transferred into a tin capsule and measured for nitrogen and carbon stable isotope composition using a Delta V Plus Isotope Ratio Mass Spectrometer, coupled with a Flash 2000 Elemental Analyzer via continuous flow. Stable isotope ratios were expressed as δ, i.e., as the deviation in per mille (‰) from an international standard—PDB (Pee Dee Belemnite) for carbon and atmospheric nitrogen for nitrogen. International reference material was measured for calibration and measurement precision, which was < 0.1‰ for both carbon and nitrogen. To test for sample homogeneity, 10 hair subsamples were measured twice. The average difference between the repeats was 0.195‰ for δ^13^C and 0.057‰ for δ^15^N. We also made 23 measurements of the standards in between the sample measurements, and calculated the difference between each measurement and the certified value of the standard. The average difference between these values was 0.072‰ for δ^13^C and 0.031‰ for δ^15^N. The size of the isotopic niche in Ukrainian dogs from the five sampling sites was estimated using Standardised Ellipse Area, corrected for differences in sample sizes. This analysis was carried out using the SIBER package (Jackson et al. 2011).

The isotopic signatures from hair samples were compared with the signatures of other canids: (1) dogs from Ukraine dated from classic antiquity to the Middle Ages (5th century BC–2nd AD) (Grandal‐d'Anglade et al. 2021) (*N* = 23, bone samples), (2) Palaeolithic dogs/wolves from Ukraine dated between 13,500 and 14,600 ^14^C yBP (Drucker et al. 2018) (*N* = 5, bone samples), (3) modern dogs from the UK (Bol and Pflieger 2002) (*N* = 4, hair samples), (4) modern Eastern European wolves (Pilot et al. 2012) (*N* = 110, muscle tissue samples), (5) historical French wolves from 1878 to 1915 (Doan et al. 2023) (*N* = 3, bone samples), (6) historical Finnish wolves from the 1880s (Junno et al. 2024) (*N* = 7, hair samples) and (7) historical Finnish wolves from the 1880s that have been reported to kill and feed on humans (Junno et al. 2024) (*N* = 3, hair samples).

The trophic discrimination factor (TDF) between diet and tissue strongly varies between tissue types. Hair and muscle tissues are depleted in ^13^C relative to bone collagen, while these differences in ^15^N are negligible (Yeakel et al. 2009; Nardoto et al. 2006; Roth and Hobson 2000). Using specific TDF values for carnivorous mammals, we adjusted δ^13^C values of muscle tissue by +1.3‰ and collagen by −1.3‰ to be comparable to hair (Stephens et al. 2023), given that our target samples represented this type of tissue. In addition, we accounted for the global decrease of ^13^C in atmospheric CO_2_ over the last 150 years (SUESS effect) by adjusting the δ^13^C values according to a ^13^C SUESS correction model (Dombrosky 2020). We also adjusted the δ^13^C isotope ratios in the Late Pleistocene dogs by −0.3‰, following Leuenberger et al. (1992).

### 2.6 Social Group Size

The differences in the social group size between the regions were assessed based on 381 photographs showing 489 stray dogs. These photographs were taken independently of the phenotypic data and hair sample collection described above, were focused on stray dogs only and were based on the same protocol in all regions where they were taken (Figure 2). Persons collecting the photographs were instructed to photograph any stray dogs encountered, alone or in groups, and to include in the photographs all the dogs from a group. Based on these photos, we assessed the distribution of the group sizes in each of the three zones.

### 2.7 Statistical Analyses of Phenotypic and Isotopic Differentiation

Inter‐group differentiation in phenotypic traits with continuous numerical values (weight, height, BMI, proportion of leg length to body height) and isotopic data (δC^13^ and δ^15^N values) was tested using the Kruskal‐Wallis test and post hoc Dunn's test. For traits with normal distribution, we additionally used ANOVA and post hoc Tukey test. To test for the normality of the distribution we used the Anderson‐Darling test.

For morphological traits assessed as categorical variables, we assessed the pairwise correlations using Spearman's rank correlation and tested their significance using the cor.test function in R (R Core Team 2021). To test for differentiation among dogs from the FL, DT and PST groups in these morphological traits, we carried out permutational multivariate analysis of variance (PERMANOVA) (Anderson 2001) and nonmetric multidimensional scaling (nMDS) ordination based on Bray–Curtis dissimilarities. All morphological traits except tail shape were analysed together. Tail shape was analysed separately, because data for this trait were available for a considerably smaller number of individuals. Differentiation in age and disease occurrence was tested in a separate analysis as well. It should be stressed that these analyses were based on individual data rather than mean values for each zone. For details, see Data S1.

In addition, we carried out an individual‐based spatial analysis of the effect of war‐related environmental damage on the BMI of dogs across Ukraine. For this purpose, we reconstructed a military activity intensity map, based on the data from the VIINA 2.0 database (Zhukov 2023). This database collects georeferenced reports on violent incidents in Ukraine during the war from Ukrainian and Russian media, classified into standardised categories. We included only the categories characteristic of front‐line conflict zones (Table S3), and removed those deemed non‐relevant (e.g., “Cyberattacks, including DDOS, website defacement”). We used QGIS to calculate the number of events from all the included categories in a 50 km radius buffer zone surrounding each sampled dog. We used a relatively large buffer, because many dogs for which we obtained measurements of morphological traits were sampled in animal shelters, and therefore could originate from a broader area surrounding the shelter. We calculated the number of violent events within each buffer in the period from January 2022 (the last month before the start of the war) to January 2024 (the last month of data collection for this study).

In order to account for economic changes in rural and urban areas during the war, we used remote sensing data on vegetation (Normalized Difference Vegetation Index—NDVI) in agricultural areas and Nighttime Light Intensity (further referred to as luminosity) to estimate the intensity of agricultural land use and urban economic activity, respectively (following Zhukov 2023). The NDVI data were obtained from the Moderate Resolution Imaging Spectroradiometer (MODIS) MYD13C1 cloud‐free spatial composites database from NASA Earth Observation Data (Didan 2021). To focus on agricultural areas, we cropped the NDVI data to include only land used for farming (Table S4) using a land‐use classification mask derived from OpenStreetMap (April 2022). We also obtained the NDVI data from all types of land within a 50 km radius, to provide a broader context for vegetation changes. The luminosity data were obtained from VIIRS (Visible and Infrared Imaging Suite) on the JPSS (Joint Polar‐orbiting Satellite System) (Elvidge et al. 2021). To quantify the changes, we collected the data on these variables from both January 2022 and January 2024, and calculated the difference, which served as a measure of economic changes in rural and urban areas during the war. Finally, to take environmental variation across the study area into account, we obtained data on climatic and environmental variables from the WorldClim database (www.worldclim.org), focusing on variables that could potentially affect dogs' phenotypic traits: mean annual temperature and precipitation, isothermality, and elevation. These data were collected with 2.5° resolution.

We used Generalised Linear Regression to estimate the spatial correlation between the BMI and the explanatory variables described above. A spatial distribution map of the BMI was produced by carrying out an Inverse Distance Weighting interpolation in ArcGIS Pro, including only dogs with the BMI measured (for justification of the choice of the spatial interpolation method, see Data S1). Interpolations were carried out with the following settings: output Cell Size: 0.15; Power: 5; Number of points 20 for traits with reduced sample sizes, and 30 for traits found in most sampling locations. The same interpolation was carried out for the number of violent events, luminance difference, and NDVI difference for agricultural areas as well as for the total vegetation. These spatial distribution rasters were then subsampled by creating a grid of 40 rows by 70 columns covering the country extent. The raster values for each variable were then added to the center point of the squared grid, using the “Extract Multi Values to Points” tool. All variables were then scaled in R, using the scale() function, after which they were re‐imported into ArcGIS Pro. The effect of the explanatory variables on the dog BMI was tested using the Generalised Linear Regression tool implemented in ArcGIS Pro. An initial analysis was done to test for correlation between explanatory variables. The GLR analysis was then repeated using only non‐correlated explanatory variables.

## 3 Results

### 3.1 Dogs Living Close to the Front Line Are Smaller and Have Lower BMI Compared With Those From Other Regions

Height of the dogs from the entire study area ranged from 14 to 110 cm, with an average of 45.32 cm and a median of 45 cm (*N* = 375). The weight ranged from 1 to 70 kg, with an average of 19.13 kg and a median of 18 kg (*N* = 616). Small dogs (< 40 cm) and those of medium height (40–60 cm) occurred with similar frequencies (42% and 43%, respectively), while the proportion of large (60–90 cm) and very large dogs (90–110 cm) was 14% and 1%, respectively.

The BMI ranged between 0.48 and 4.62, with an average and median of 2.42 (*N* = 367). Height was positively correlated with BMI (*R* ^2^ = 0.077, *p* < 0.0001), and individuals with the lowest BMI (0.48–1.00) were considerably smaller (28.2 cm on average) than those with the highest BMI (4.00–4.62; average height 51.6 cm). Only five dogs (1.3%) had a BMI lower than 1, 26% of dogs had values between 1 and 2, 50% between 2 and 3, and 23% had values larger than 3. All five dogs with a BMI below 1, indicative of malnutrition, had small body sizes, with the height ranging between 20 and 35 cm.

**FIGURE 3:**
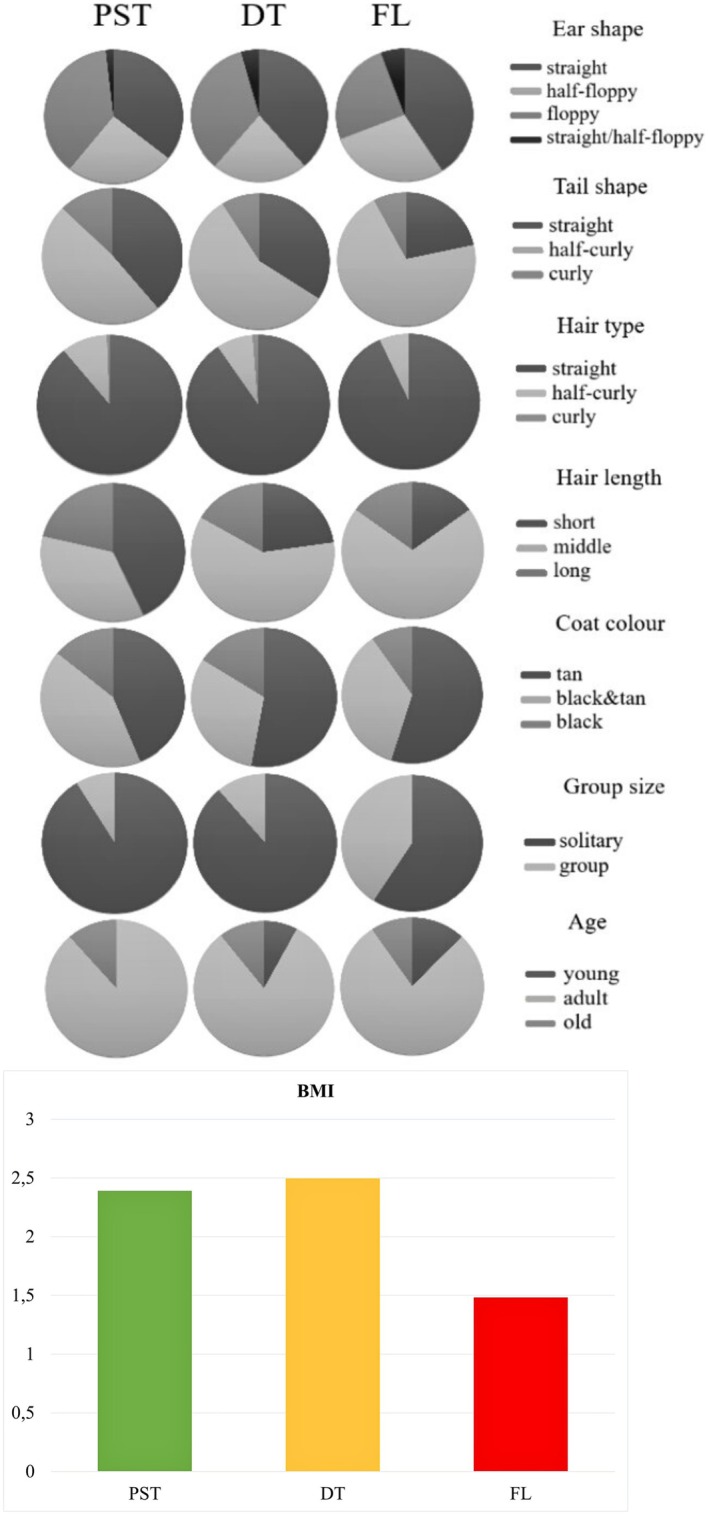
Distribution of phenotypic traits in dogs grouped according to the sampling zone: BMI, body mass index; DT, dangerous territories; FL, front line; PST, potentially safe territories. The mean values for each zone are shown here to illustrate the differences between them, but the statistical analyses were carried out based on individual values.

There were considerable differences in height, weight and BMI between dogs from the PST, DT and FL zones (Table 1, Tables S5–S10, Figure 3). In FL and PST, small dogs predominated (20–40 cm), and the average height was 39.8 and 37.9 cm, respectively. In DT, medium‐size dogs (40–60 cm) predominated and the average height was 50.7 cm. We found statistically significant differentiation between the three zones in the height distribution (Kruskal‐Wallis test, χ^2^ = 31.4, *p* = 1.52e‐07, *N* = 301). This differentiation was significant between two pairs of zones, FL–DT and PST–DT, but non‐significant between PST and FL (Tables S9 and S10).

**TABLE 1:** Summary of the significant differences between the phenotypic traits of dogs from the three zones in Ukraine delimited based on the intensity of war activities: DT, dangerous territories; FL, front line; PST, potentially safe territories. The comparisons between the zones were carried out based on individual data rather than mean values (see Methods). Significant results are marked in bold. ***p ≤ 0.001, **p ≤ 0.01, *p < 0.05.

Type	Features	All zones	FL‐DT	FL‐PST	DT‐PST
Genetically determined traits (binominal data)	Snout and ears shape, hair length and structure, coat colour	0.001***	0.01**	0.001***	0.001***
	Tail shape	0.006**	0.48	0.008**	0.008**
Time‐varying traits (binominal data)	Age	0.029*	0.71	0.084	0.037*
	Disease	0.38	0.031*	0.68	0.042*
Body measurements (continuous data)	BMI	3.7e‐05***	0.00026***	0.018*	0.017
	Height	1.5e‐07***	1.6e‐02***	1.00	5.1e‐07***
	Weight	0.0061**	0.012*	0.073	0.29
	Leg proportion	0.0018**	0.035*	0.74	0.0088**
Counts (continuous data)	The size of dog groups	0.021*	0.03*	0.005**	0.294
Stable isotope measurements (continuous data)	δC^13^ values	9.5e‐05***	0.00076***	0.25	0.0065**
	δ^15^N values	0.0017**	0.0012**	0.027*	0.54

In FL, dogs of small weight (6–10 kg) were the most frequent class and the average weight was 13.6 kg. In both DT and PST broader weight ranges were observed and as a result the average weight was higher (20 and 17.7 kg, respectively). We found statistically significant differences in weight distribution between the three zones (Kruskal‐Wallis test, χ^2^ = 10.2, *p* = 0.006, *N* = 541). The pairwise differentiation was significant between one pair of zones only, DT and FL (Table S9).

**FIGURE 4:**
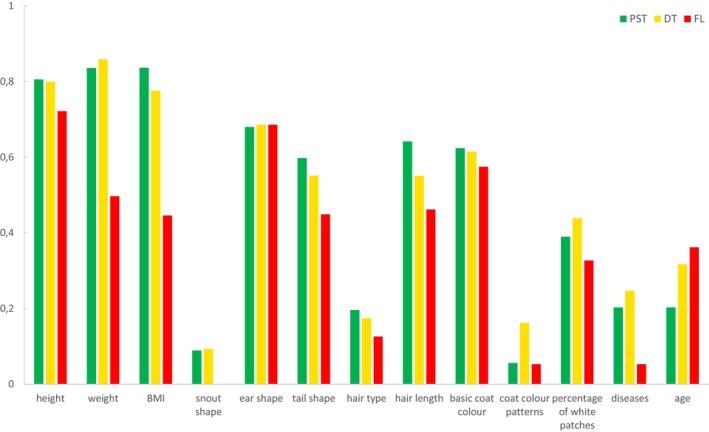
Comparison of diversity within each phenotypic trait between dogs from the three zones in Ukraine: DT, dangerous territories; FL, front line; PST, potentially safe territories. The diversity was measured as 1‐D, where D is the Simpson's index.

Average BMI in FL (1.48) was considerably lower than in DT (2.50) and PST (2.39). We found statistically significant differences between the three zones in the BMI distribution (Kruskal‐Wallis test, χ^2^ = 20.4, *p* = 3.72e‐05, *N* = 293), and all pairwise comparisons between zones were significant (Tables S9 and S10). Height, weight and BMI, presented as frequencies of categorical values, showed a decrease in diversity (measured with Simpson's index) in FL compared with DT and PST (Figure 4 and Table S11).

The proportion of dogs with short legs (i.e., with leg length of less than 50% of the body height) in the entire population was 14%, and a declining trend was observed for this trait in the west–east gradient, from 18% in PST to 9% in FL (Table S12). We found statistically significant differences between the three zones in the distribution of the proportion of leg length to body height (Kruskal‐Wallis test, χ^2^ = 12.67, *p* = 0.0018, *N* = 206).

### 3.2 The Correlation of BMI With the Frequency of Violent Events Cannot Be Explained by the Effect of Environmental Variables Unrelated to War Events

**FIGURE 5:**
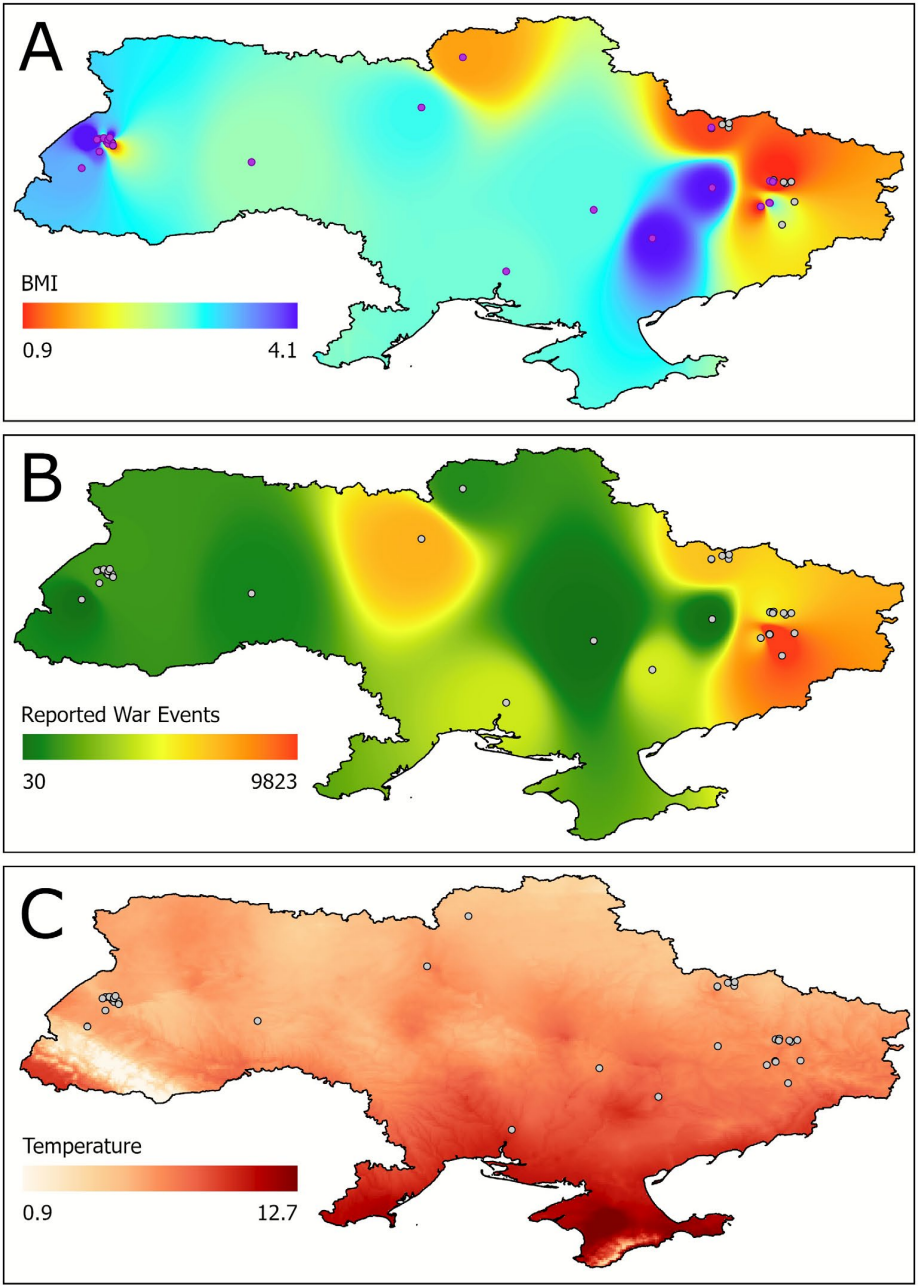
Spatial distribution of (A) interpolated BMI values of the dogs studied; (B) interpolation based on sum of violent events and (C) mean annual temperature across Ukraine.

The map created by interpolation of the BMI values from individual dogs into the entire territory of Ukraine showed sharp differences between dogs sampled at the front line and those sampled in the neighbouring areas to the west (Figure 5A). This map shows a very good correspondence with the differences in the number of violent events reported for the front line and the areas to the west (Figure 5B). Moreover, the distribution map of violent events shows distinct patterns for western, central and eastern Ukraine, closely coincident with the defined division into PST, DT and FL zones. The FL and PST zones have the highest and lowest number of violent events, respectively. The DT area shows a larger spatial variation in the number of events; therefore supporting our qualitative assessment that it is of intermediate risk (Figure 5B).

Luminosity as well as NDVI in both the agricultural areas and the total land area showed substantial differences between January 2022 and January 2024 (Figures S1 and S2). In particular, the spatial changes in luminosity over time reflect population displacement, with a noticeable shift westward and depopulation in areas close to the front line. Both luminosity and NDVI showed a strong correlation with the number of violent events (War Events, Figure S3), therefore we excluded them from the final analysis and considered the number of violent events to accurately reflect changes in human activity due to the war effort. Furthermore, all environmental variables were strongly correlated with the mean annual temperature (Figure S3). Isothermality was an exception, but this variable was correlated with precipitation and elevation. Therefore, the GLR models were all done using two explanatory variables: the number of war events reflecting the temporal changes to human activities due to the war, and mean annual temperature reflecting geographic differences in natural environmental factors.

**FIGURE 6:**
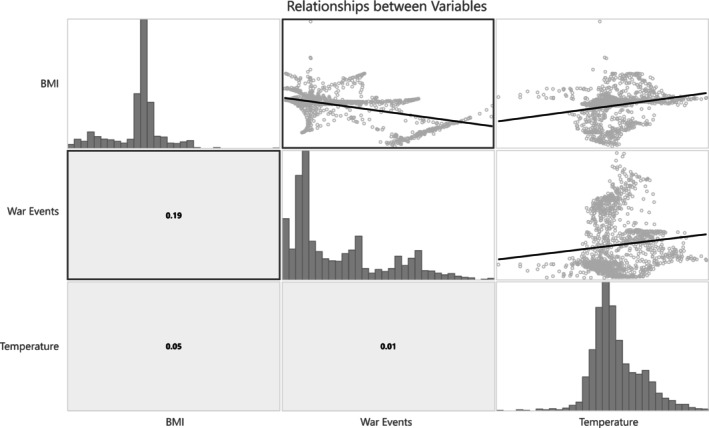
Relationship between dog BMI and the two explanatory variables: the number of violent events and temperature, assessed using the Generalised Linear Regression model. In the lower left panels, the *R* ^2^ values for the respective pairwise correlations are shown.

The BMI showed a significant negative correlation with War Events (GLR coefficient = −0.46, *p* < 0.000001) and a weaker but significant positive correlation with temperature (GLR coefficient = 0.27, *p* < 0.000001; Figure 6 and Table S13). Temperature shows an increasing trend along a northwest–southeast gradient (Figure 5C) and a weak positive correlation with War Events (*R* ^2^ = 0.01). Therefore, the significant BMI decline with the increase in War Events cannot be explained by an effect of temperature or other climatic variables.

### 3.3 Frequencies of Most Common Phenotypes Increase Along the West–East Cline

For 722 out of 763 dogs studied, including 174 from PST, 473 from DT and 74 from FL, we obtained photographs of sufficient quality to assess all or most of the morphological traits: snout shape, ear shape, tail shape, hair type, hair length, and coat colour patterns (Tables S14–S20). Categories within each trait are described in the Methods section.

For 395 dogs, for which we obtained a complete set of all these morphological traits, we analyzed the pairwise correlations between the traits. We found strong correlations between hair type and hair length, between snout shape and both hair type and hair length, as well as between the presence of coat color patterns (e.g., brindle) and the proportion of white patches on the coat. These variables were significantly correlated after Bonferroni correction accounting for multiple comparisons (Table S21). The traits considered as part of the Domestication Syndrome (Wilkins et al. 2014; see the discussion), that is, snout shape, ear shape, tail shape, and the presence of white patches, showed no significant pairwise correlations with or without Bonferroni correction. In the analysis carried out for each zone separately, we found a correlation between ear shape and the percentage of white patches in the PST zone, which was significant in the individual pairwise comparison but non‐significant after Bonferroni correction (Table S21).

For 700 dogs, we obtained a complete set of phenotypes for all of the morphological traits except tail shape, which was therefore analyzed separately in the following analyses. Phenotypes that occurred with the highest frequency in the entire population were as follows: mesocephalic snout shape (96% of dogs), straight ears (38%), straight hair (90%), medium‐length hair (56%) (Figure S4). Each of these traits showed an increase in frequency from PST to FL, with intermediate values in DT (Table S14; Figure 3). Thus, the traits that already predominated in the entire population became even more frequent in the FL dogs.

Coat colour types (see Table S1) occurring with the highest frequency in the entire population were as follows: tan basic coat colour (50%), absence of additional colour patterns (93%) and less than 20% of white patches (75%). Tan colour showed an increasing trend from PST through DT to FL (from 43% to 54%). Additional colour patterns did not show clear trends except for brindle, which showed an increase from absence in PST to 1% in FL; this could, however, occur by chance. The number of dogs with < 20% of white patches changed from 77% in PST to 81% in FL, but without a clear trend, as the lowest value occurred in DT (Table S15).

The nMDS ordination based on Bray–Curtis dissimilarities showed limited partitioning of the phenotypic traits described above between the FL, DT and PST zones (Figure S5). Nevertheless, we found significant differentiation among the zones (PERMANOVA, *R* ^2^ = 0.016, *F* = 5.80, *p* < 0.001, *N* = 700; Table S16). The differences explained a small proportion of the total variation, because of the large variation observed within each zone for each trait assessed. The post hoc pairwise tests showed significant pairwise differentiation between all zone pairs (Table S17). Traits that showed the greatest contribution to the differentiation between zones were coat colour, hair length and ear shape, and the specific phenotypes contributing the most to the differentiation were tan‐and‐black and tan coat colour, middle hair length and straight ears (Table S18).

The tail shape had a larger number of missing data than other traits; therefore we analysed it separately for smaller sets of dogs (*N* = 305). The predominant tail shape in the entire population was half‐curly, which occurred in 57% of individuals and showed an increasing frequency from PST (48%) to FL (71%), while both straight and curly shapes showed a reverse trend (Table S19 and Figure 3). Differences in frequencies of different tail shapes among the three zones were statistically significant (PERMANOVA, *R* ^2^ = 0.025, *F* = 3.88, *p* = 0.006). The post hoc pairwise tests showed significant pairwise differentiation between two zone pairs (DT‐PST and FL‐PST), but no significant differentiation between FL and DT (Table S20).

Snout shape, tail shape, hair type and length, basic coat colour, additional colour patterns and the proportion of white patches showed a decrease in diversity (assessed using the Simpson's index) in FL compared with DT and PST (Table S12 and Figure 4). The ear shape was the only phenotypic trait among those assessed that did not decrease in diversity in FL.

### 3.4 Dog Population From the Front Line Has Larger Proportion of Young Individuals and Smaller Proportion of Individuals With Diseases

We used the photographs to assign dogs to three age categories: young, adult, and old. In the total population studied, 83% of individuals were adults and the frequency of old individuals was higher than young ones (11% vs. 6%). The frequencies of adult and old dogs showed a declining trend from PST to FL, and the frequency of young dogs showed the reverse trend (Table S22). The diversity index for age categories showed an increasing trend from PST to FL (Table S12), which was due to an increasing number of young individuals, leading to more balanced proportions of the three categories. Differentiation among FL, DT and PST populations for the age class distribution, measured using Bray–Curtis dissimilarities, was statistically significant (PERMANOVA, *R* ^2^ = 0.0085, *F* = 2.97, *p* = 0.029, *N* = 700; Table S23). However, the post hoc pairwise tests showed significant differentiation only between DT and PST (*p* = 0.037), while the differentiation between other pairs of zones was non‐significant (Table S24).

In the total dog population, 12% of dogs had visible diseases or injuries, which were classified into broad categories: leg injuries or disabled/missing legs (occurring in 31% of all individuals with injuries/diseases), eye diseases and eye loss (25%), wounds and scars (16%), skin diseases (5%), and other diseases that were noted in one or a few individuals only (22%; see Methods). One individual had injuries directly caused by military actions (bullets in the body).

All disease categories displayed the lowest proportions in FL (Table S22). Overall, only 3% of FL dogs had visible diseases, while this proportion was 13.5% in DT and 11% in PST. The proportion of “other diseases” may be biased, because some of them could only be diagnosed by a veterinarian, which could only be done for shelter dogs. However, after the exclusion of this category, the trend remained the same and the percentage of FL dogs with diseases was reduced to 1.4% (all of which were leg injuries). We calculated the diversity index, based on different categories of injuries/diseases and a category of individuals without detected diseases. We found no clear trend in disease diversity along the west–east gradient, but the diversity in FL was the lowest (Table S12 and Figure 4).

Differentiation among FL, DT and PST populations for disease occurrence, measured using Bray–Curtis dissimilarities, was non‐significant (PERMANOVA, *R* ^2^ = 0.0033, *F* = 1.17, *p* = 0.38, *N* = 700; Table S25). However, the post hoc pairwise tests showed significant pairwise differentiation between two population pairs (FL–DT and DT–PST), while the differentiation between FL and PST was non‐significant (Table S26).

### 3.5 Isotopic Composition Suggests Low Trophic Level

**FIGURE 7:**
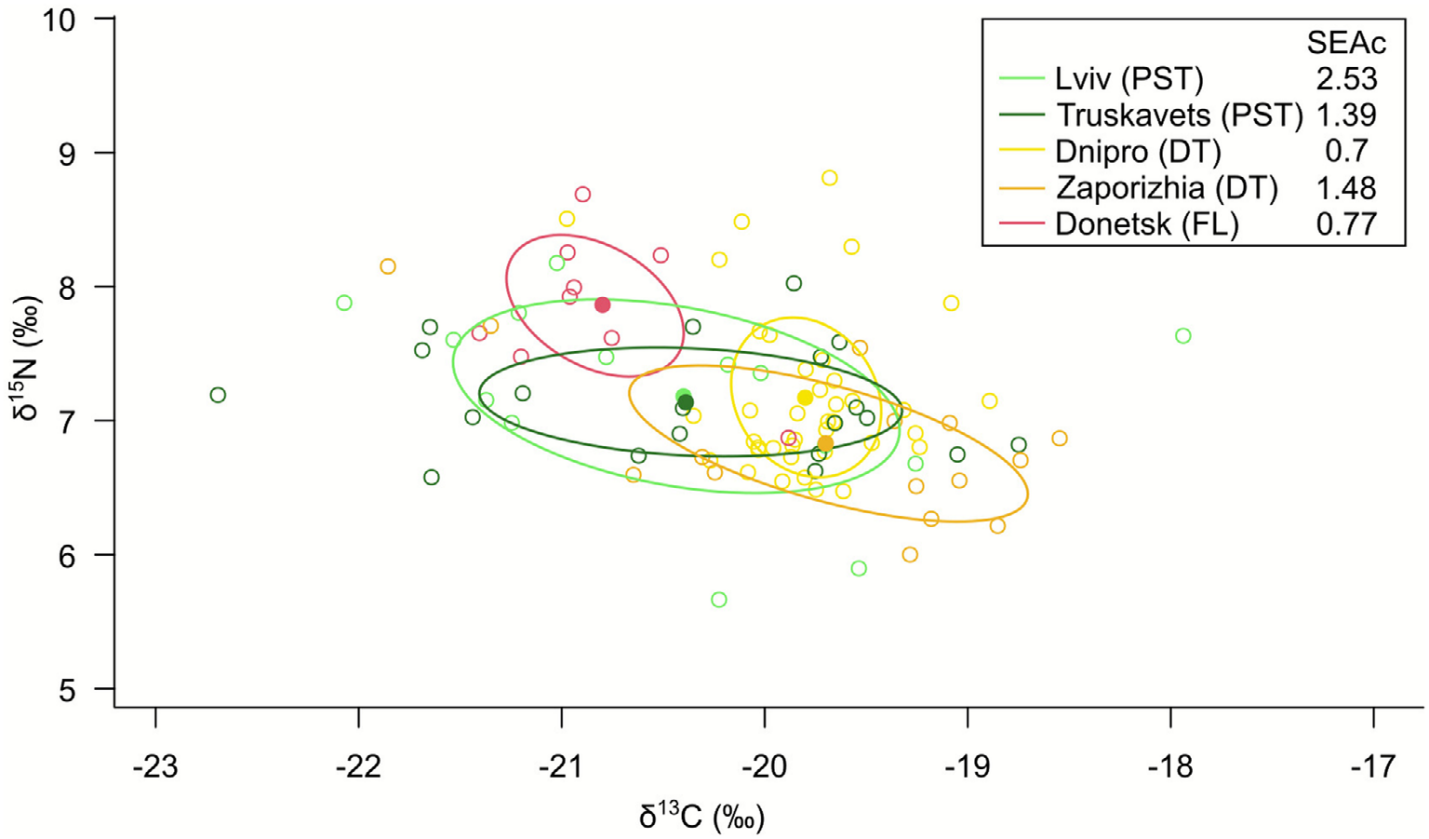
The size of the isotopic niche in Ukrainian dogs from five locations estimated using Standardized Ellipse Area, corrected for differences in sample sizes. The locations represent the three zones: DT, dangerous territories; FL, front line; PST, potentially safe territories.

We found considerable variation in the isotopic signatures of the dogs studied: δ^13^C values varied from −22.69‰ to −17.94‰, and δ^15^N from 5.66‰ to 8.81‰. Considerable variation within each sampling site was observed, as well as overlap between sites (Figure 7). When the mean value for each sampling site was considered, two adjacent locations in Lviv Oblast (PST) clustered together and two adjacent locations in Zaporizhia and Dnipro Oblasts (DT) formed another cluster (Figure S6). The location from Donetsk Oblast (FL) was distinct and had the lowest δ^13^C values and the highest δ^15^N values. The size of isotopic niches estimated using Standardized Ellipse Area, corrected for differences in sample sizes, was largest in the Lviv city (PST) and smallest in Dnipro Oblast (DT) (Figure 7). Differences in the isotopic composition between the PST, DT and FL zones were statistically significant (Kruskal‐Wallis test, δ^13^C values: χ^2^ = 18.5, *p* = 9.53e‐5; δ^15^N values: χ^2^ = 12.7, *p* = 0.0017; Table S27), but the pairwise differentiation between the zones was significant only for some pairs (Table S28).

The isotopic signatures of Ukrainian dogs were compared with published isotopic signatures of other European dog and grey wolf populations, covering periods from the Palaeolithic to modern times (Pilot et al. 2012; Drucker et al. 2018; Grandal‐d'Anglade et al. 2021; Doan et al. 2023; Junno et al. 2024). We included the isotopic signatures of modern pet dogs in order to test whether the diet of the Ukrainian dogs we studied may be similar to the diet of pet dogs elsewhere in Europe (i.e., whether they mostly consumed processed pet food that does not reflect isotopic signatures of local food sources). We only found such data from the United Kingdom (Bol and Pflieger 2002).

**FIGURE 8:**
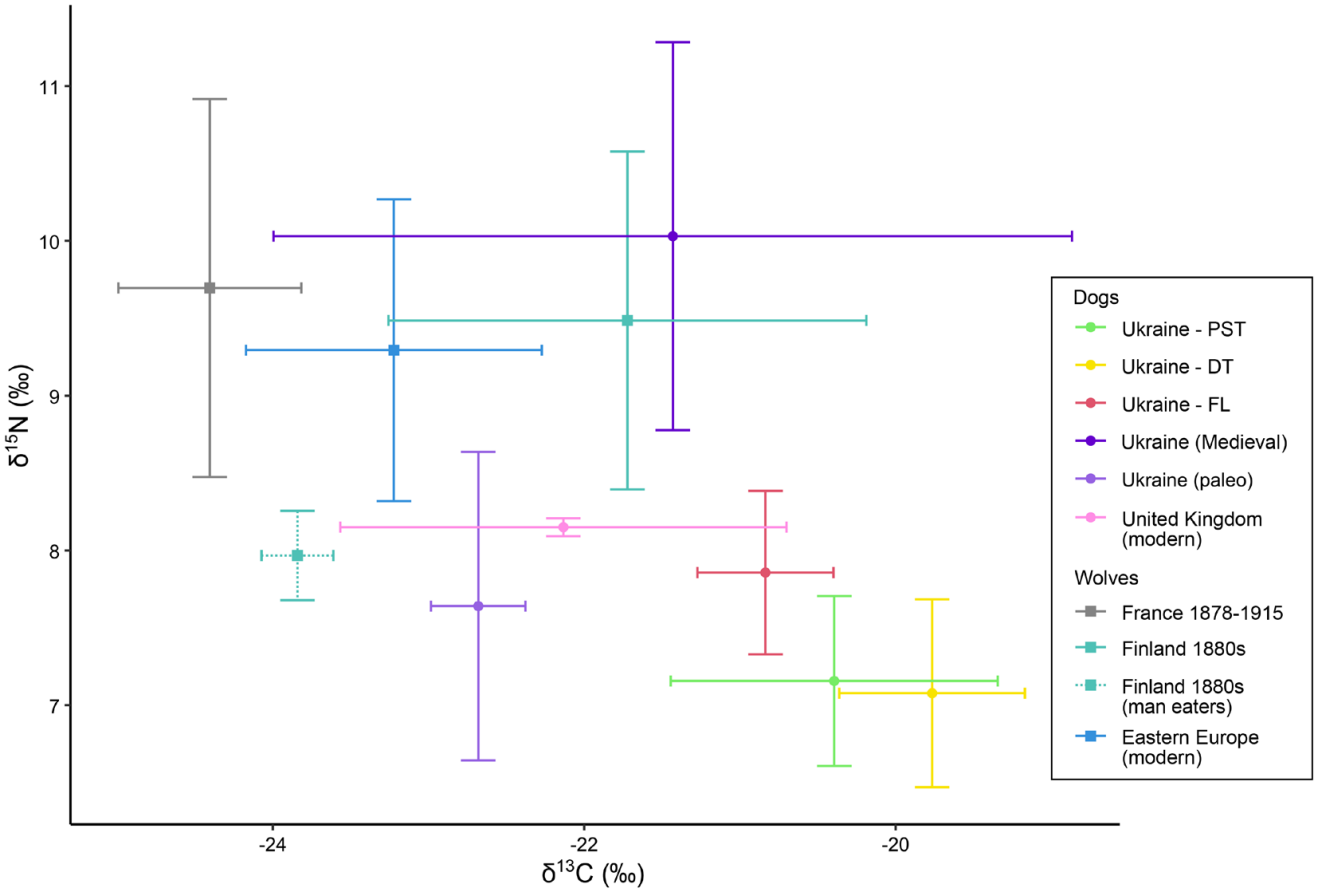
δ^13^C and δ^15^N stable isotope values of Ukrainian dogs compared with dogs and wolves from other populations (data from earlier studies). Mean and standard deviation for each group are shown. Domestic dogs, including the Ukrainian dogs studied, are shown as circles, while grey wolves are shown as rectangles. The Ukrainian dogs originated from two locations in Potentially Safe Territories (PST), two locations in Dangerous Territories (DT), and one location at the Front Line (FL).

δ^13^C values in Ukrainian dogs were high compared with most other dog and wolf populations, except the historical dogs from Ukraine dated between Classical antiquity and the Middle Ages (Grandal‐d'Anglade et al. 2021) that showed comparable values, but a broader range (Figure 8). There was a partial overlap in δ^13^C values between the Ukrainian dogs and modern pet dogs from the United Kingdom, but it resulted from a broad range of δ^13^C values observed in the latter population, which likely reflects a large diversity of primary sources of food items included in the commercial pet food. The Ukrainian dogs did not show such large variation. High δ^13^C values they display may suggest a diet rich in C4 plants or animals feeding on them.

The δ^15^N values in the Ukrainian dogs were low compared with δ^15^N values in other modern and historical dogs as well as wolves. Comparable δ^15^N values were only observed in Palaeolithic dogs from Ukraine (Drucker et al. 2018). In contrast, historical dogs from Ukraine (Grandal‐d'Anglade et al. 2021) had δ^15^N values higher by more than 2‰. When δ^13^C and δ^15^N values are considered jointly in a two‐dimensional space, Ukrainian dogs did not show close similarity to any dog or wolf population assessed (Figure 8).

### 3.6 Dogs Living Close to the Front Line Form Groups More Frequently Than in Other Regions

We used 381 photographs of stray dogs from all regions of Ukraine except Luhansk Oblast and Crimea (23 regions, Figure 2) to obtain information about the proportion of dogs occurring in social groups and the sizes of such groups. The photographs showed 489 dogs, most of which were found alone (86.9% of photographs). The groups consisted of up to 11 individuals, but most often a group consisted of 2 dogs (5.9% of photographs), 3 dogs (2.7%) or 4 dogs (1.6%).

The frequency of solitary individuals decreased along the PST‐DT‐FL gradient from 91% to 59% of photographs, while the frequencies of pairs, trios and larger groups increased (Table S29). We found significant differences in the distribution of social group sizes (including solitary individuals) between the three zones (PERMANOVA, *R* ^2^ = 0.02, *F* = 3.88, *p* = 0.021, *N* = 381; Table S30). The post hoc pairwise tests showed significant pairwise differentiation between two zone pairs (FL‐DT and FL‐PST), but no significant differentiation between DT and PST (Table S31).

Groups with maximum size were recorded in DT. In this region, one group of 7 individuals and one of 11 individuals were found. These were the largest observed groups, and both were formed by pups only. Relatedness among the pups within each group was unknown, but based on the phenotypes they do not seem to originate from one litter (Figure S7). In DT 31.8% of groups were formed by pups and 68.2% of groups were formed by adults. In PTS, 7.7% of groups were formed by pups, and the remaining 92.3% by adults, while in FL all groups were formed only by adult dogs. Females with pups were counted separately, not as groups, and their largest frequency (2.5% of all photographs) was seen in DT.

## 4 Discussion

The effects of war on domestic dogs occur both at an individual level (e.g., decline in health, food deprivation) and a population level (e.g., changes in phenotypic composition of a population and in social structure). Our approach allowed us to observe or infer some of these changes and consider their implications.

### 4.1 Changes in Frequencies of Phenotypic Traits Along the West–East Gradient Are Consistent With Natural Selection

Several phenotypic traits increased in frequency along the west–east gradient, including: mesocephalic snout shape (i.e., neither flattened nor elongated), straight ears, straight hair of medium length, regular leg length, half‐curly tail and tan coat colour with a small proportion of white patches. Most of these traits are typical for wild canids and are likely to be maintained by natural selection. The exceptions are the tail shape, which is straight in wild canids, and the coat colour, which shows high interspecific variation. It may be expected that the frequencies of these traits and the rate of their frequency changes along the gradient reflect their adaptive value for free‐ranging dogs. The traits that occurred at the highest frequencies in FL were mesocephalic snout shape (100%) and straight hair (93%), while the trait with the greatest increase in frequency along the west–east gradient (from 36% in PST to 70% in FL) was middle‐length hair.

At the opposite end of the spectrum of morphological traits typical for wild canids are the traits that are specific to domestic dogs and other domesticated mammals, such as floppy ears, curly tails, brachiocephalic snout shape, and coat depigmentation (i.e., large proportion of white patches). These traits are among the set of apparently unrelated traits common in domesticated mammals (Darwin 1868) that are considered part of “Domestication Syndrome” (Wilkins et al. 2014). There are several hypotheses on the mechanism underlying the joint occurrence of such traits in domesticated mammals, including reduced migration of neural crest cells from the neural tube during embryonic development (Wilkins et al. 2014; Sánchez‐Villagra et al. 2016), attenuated activity of the hypothalamic–pituitary–adrenal axis that modifies embryonic and early postnatal development (Trut et al. 2009), or altered sexual selection patterns and reproductive physiology (Gleeson and Wilson 2023), while some studies question the existence of the Domestication Syndrome (Lord et al. 2020).

In our study, the morphological traits considered as part of this Syndrome decreased in frequency along the west–east gradient, but we found no correlation among these traits in pairwise comparisons. However, a correlation between two Domestication Syndrome traits (ear shape and the proportion of white patches) found in the PST zone only suggests that natural selection favoring “wild‐type” traits may result in decoupling of domestication‐related traits. The observed differences between the zones in the frequencies of different phenotypes are unlikely to result from the selection against one of several co‐varying traits and may instead reflect the adaptive value of each “wild‐type” trait for free‐ranging dogs under strong selective pressures.

For example, modified snout shape is associated with health problems; brachycephalic dogs experience severe respiratory disorders, poor tolerance of physical exercise, significant heat sensitivity and sleep problems (Roedler et al. 2013), therefore this trait is likely to be subject to purifying selection in free‐ranging dogs. Short legs may cause less efficient walking and running, which may have important consequences for avoiding danger and finding food sources. Hair shape and length may affect thermoregulation (Jørgensen et al. 2020; Mota‐Rojas et al. 2021) and ectoparasite load (Murray 1987; Pagel and Bodmer 2003), therefore the prevalence of the middle‐length hair may result from a trade‐off between these two selective pressures. Therefore, these traits are likely to have a strong and immediate effect on fitness in stray dogs. Ear shape may affect hearing, ear infection rate, and thermoregulation (Degen 2012). Many dogs at the front line were partially or completely deaf as a result of acoustic trauma. The trade‐off between advantages and disadvantages of pointed ears may explain moderate frequency changes of the ear shape between the three zones. The fitness effect of the tail shape may be associated with intraspecific communication as well as communication with humans. Half‐curly tails may be optimal for displaying the wagging behaviour, which may facilitate positive interactions with humans (Leonetti et al. 2024).

The fitness effect of coat colour is not entirely clear. Wild canids show considerable interspecific variation in coat colour, and some widely distributed species like the grey wolf also show intraspecific variation (Gipson et al. 2002; Castelló 2018). Tan colour is common across modern dog breeds originating from diverse regions and predominates in Australian dingoes and feral or semi‐feral dog populations, such as Carolina dogs (Brisbin and Risch 1997; Newsome et al. 2013; Bannasch et al. 2021; Cairns et al. 2021), which suggests a selective advantage of this colouration. Coat colour‐associated mutations have pleiotropic effects on sensory systems, behavioural traits as well as reproductive and immune systems (Reissmann and Ludwig 2013), therefore the fitness effect of coat color may be indirect. For example, white patches result from altered melanogenesis (melanin production), which can also affect endocrine and nervous systems as well as skeletal development (Brancalion et al. 2022).

Significant changes in the frequencies of heritable phenotypic traits along the PST‐DT‐FL gradient, the correlation of these traits with the frequency of war‐related violent events as well as the increase in the frequencies of the most common phenotypes collectively suggest that these traits may be under strong natural selection. This is further supported by the decrease in the diversity of most of these traits along the same gradient, consistent with increased strength of natural selection close to the front line. The period studied was too short for natural selection to occur via differential reproductive success; therefore, it is more likely to be based on differential mortality and—to some extent—emigration. This implies that the strong environmental changes caused by the war may trigger differential survival dependent on certain phenotypic traits.

Rapid evolutionary shift resulting from military activities has also been demonstrated in African savannah elephants ( *Loxodonta africana* ). During the Mozambican Civil War, intense poaching carried out by armies on both sides of the conflict resulted in strong selection favoring tusklessness in females, with persistent, hereditary change observed in the post‐war population (Campbell‐Staton et al. 2021). In that case, the selective pressure was associated with deliberate hunting targeting high‐value ivory. In contrast, our study presents the case of a rapid change in frequencies of morphological traits likely resulting from an impact of military activities.

### 4.2 Dogs at the Front Line Have Small Body Size and Low BMI

Body size is an adaptive trait, and there is a trade‐off between the advantages of smaller size (less nutrition required for survival) and larger size (better resource competition abilities). Small and medium sizes were the most common among the dogs studied (85% of individuals), suggesting that large body size may be disadvantageous because of larger food requirements or other reasons (see below).

The distribution of dog sizes differed considerably between the three zones. In FL and PST, small dogs predominated, but the average weight in FL (13.6 kg) was considerably lower than in PST (17.7 kg). The DT population had the highest average body height and weight. Accordingly, the average BMI in FL (1.48) was considerably lower than in DT (2.50) and PST (2.39). This suggests that the survival rates of small dogs at the front line are higher compared with larger dogs, but nevertheless their condition is worse compared with dogs from other regions. According to observations at the front line, tall dogs die more often due to the system of mines triggered by a wire stretched above the ground at a certain height, while short dogs can pass under the wire without activating mines. Larger dogs also have a greater chance of getting killed or wounded by shrapnel from mines. Therefore, smaller body sizes of dogs at the front line could help avoid danger, but the low BMI values of these dogs point to poor body condition due to poor nutrition.

The distribution map of BMI values across Ukraine (Figure 5A) shows strong differentiation between dogs from the front line and dogs from the adjacent regions, which closely mirrors the differences in the number of violent events between these regions (Figure 5B). The significant negative correlation between dog BMI values across Ukraine and the number of violent events could result from direct effects of these events combined with reduced food availability resulting from the reduced economic activities in urbanised areas and the reduced land use for agriculture. These three explanatory variables are highly correlated and therefore individual effects of each of them cannot be differentiated. It was clear, however, that the reduction of dog BMI at the front line is not due to natural factors such as climate differentiation, because BMI was positively correlated with mean annual temperature, which increased from northwest to southeast (Figure 5C).

Although only a small proportion of dogs in the entire population (1.3%) showed clear signs of malnutrition (BMI < 1), 14% of individuals had BMI below 1.5. In DT and PST populations, this proportion was 7% and 19%, respectively, but in the FL population it was 82%. This shows that most dogs living close to the front line experience severe food scarcity, but in other regions this affects only a minority of dogs. Changes in land use are also seen in regions more distant from the front line; however changes in food supplies occurring during the war could have been associated with a decreased quality, rather than quantity, of food, including reduced access to animal protein.

### 4.3 Hair Isotopic Composition of Dogs at the Front Line Is Consistent With Malnutrition

We have no information about the isotopic composition of food sources for the dogs studied. Therefore, we based our interpretation on: (1) comparing our results with literature values of domestic dogs (as a reference for dog food consumers) and wolves (as a reference for a mainly carnivorous diet); (2) comparing dogs from different study areas and applying well‐known gradients of isotopic variation, such as C3/C4 plants for δ^13^C (Farquhar et al. 1989) and trophic position for δ^15^N (Post 2002). Ukrainian dogs from all three zones were at a low trophic level, as indicated by low δ^15^N values compared to other dog populations. Historical Ukrainian dogs (which represented a broad temporal range from antiquity to the medieval period, originated from various geographic locations across Ukraine, and most likely opportunistically used food resources available locally to humans; Grandal‐d'Anglade et al. 2021), had higher δ^15^N values, comparable with European wolf populations, suggesting a high trophic level. These contrasting δ^15^N values imply that the diet of modern Ukrainian dogs during the war is considerably different from the diet of other dog populations in Ukraine and elsewhere, both historical and modern. Low δ^15^N values compared with other dog populations may indicate an increased proportion of plant‐based food in the diet. It is possible that with the economic crisis resulting from the war, humans dispose of less meat that could then be consumed by dogs, and increase the proportion of plant‐based food in meals offered to dogs (e.g., bread, potatoes, cereals).

Relatively high δ^13^C values in Ukrainian dogs compared with other populations are more difficult to interpret, because the types of diet leading to an increase in δ^13^C values may vary, and may involve increased consumption of C4 plants (e.g., millet and corn) or increased consumption of prey species with more C4 plants in their diet (e.g., pigs or wild boar feeding on corn and corn‐based products). Historical Ukrainian dogs also had high δ^13^C values compared with other European dog populations, which was explained by high consumption of millet either directly by dogs or by other domestic animals, with the remains of food consumed by humans being given to dogs (Grandal‐d'Anglade et al. 2021). Therefore, high δ^13^C values could have resulted from a high share of C4 crops in this relatively dry and warm region of Europe in both historical and modern times.

Although Ukrainian dogs from all zones had low δ^15^N values and high δ^13^C values compared to other dog populations and to wolves, dogs from the Donetsk region (FL) had relatively higher δ^15^N and lower δ^13^C values than Ukrainian dogs from other regions. This could be the result of severe malnutrition or starvation, which in inert tissues such as hair manifests itself in elevated δ^15^N values and sometimes in a simultaneous decline in δ^13^C values (Lee‐Thorp et al. 1989; Mekota et al. 2006, 2009; Doi et al. 2017; Yeakel et al. 2009). However, the increase in δ^15^N and decline in δ^13^C values make these dogs' isotopic signature closer to that of other modern dog populations as well as to wolves, which could be also achieved by increased consumption of meat obtained via hunting and scavenging. These two potential interpretations of the isotopic differences between the FL zone and other parts of Ukraine (i.e., malnutrition vs. attempts at hunting and scavenging) are not mutually exclusive, because malnutrition may result in switching to alternative feeding strategies. For example, in the documented cases of carnivoran mammals feeding on humans, this was preceded by periods of malnutrition or starvation (Junno et al. 2024; Yeakel et al. 2009).

Dogs have been reported to feed on human corpses, usually in circumstances where a dog is trapped at home with a deceased human without food for a prolonged period (Erkol and Hösükler 2018). There have been media reports of such behaviour being displayed by stray dogs during wars. According to our knowledge, this has never been documented in scientific studies, but given that scavenging is a natural behaviour in canids, such reports cannot be dismissed. During our study, three cases of dogs feeding on human corpses in an open area have been observed at the front line. These dogs were Laika‐type (spitz‐type dogs, with wolf‐like body proportions), avoided humans and were aggressive when approached; therefore they could not be sampled, but drone footage showing one such dog is available (see Video S1).

Dogs sampled for the stable isotope analysis, including those from the front line, had very low δ^15^N values, implying that human corpses are not among their important food sources. Accordingly, low consumption of meat by these dogs implied by their isotopic signatures shows that it is unlikely that these dogs have a significant impact on wildlife. However, dogs that were sampled in this study were tame enough to be approached by humans and be subject to sampling. Individuals who approach humans are more likely to use anthropogenic food as the main dietary component. Many dogs at the front line and in de‐occupied areas avoided humans, and it is likely that they obtained food mainly by scavenging and hunting. It is therefore possible that two populations of dogs characterised by different feeding strategies may coexist at the front line, and only one of them is represented in our dataset. Feralised dogs can be studied with methods developed for wild carnivores, but such methods cannot be safely implemented during the war.

### 4.4 Composition of the Front‐Line Population Suggests High Mortality of Old and Ill or Injured Individuals

The frequencies of adult and old dogs showed a declining trend from PST to FL, and the frequency of young dogs showed a reverse trend. The decline in the frequency of old individuals was probably due to a reduced survival rate because of increased food competition and lack of veterinary care. The likely reason for the increase in the frequency of young individuals was the lack of human control over dog reproduction. The increased frequency of young individuals and decreased frequency of old ones result in a faster generation turnover, which may accelerate the process of natural selection.

The frequencies of dogs with visible diseases or injuries were considerably higher in DT and PST (13.5% and 11%, respectively) than in FL (3%). Only one individual had injuries resulting unambiguously from the war (bullets in the body), while all other injuries and diseases observed could have other origins. Leg injuries were the most frequent health problem across all zones and the only frequent health problem in FL. This suggests that either this is the most common injury in general or individuals with such injuries have better survival rates compared with other injuries or diseases. Diseases frequent in PST and DT, such as eye diseases, skin diseases, wounds and scars were not observed in FL. This may result from either lower incidence of injuries and disease in this population (which is unlikely), or low survival of ill or injured individuals. Given very limited access to veterinary care in FL, it is likely that mortality rates from diseases and injuries are high. In the longer term, high mortality is likely to intensify selection pressure against hereditary disorders and reduced immune response.

### 4.5 Dogs at the Front Line Occur in Groups More Frequently, Which May Enhance Survival

Studies on free‐ranging dogs from across the world showed that they can form social groups of varying sizes and stability (Bonanni and Cafazzo 2014). Almost 87% of photographs of stray dogs from Ukraine showed single individuals, which suggests that they either rarely formed social groups or spent a lot of time apart from their groups. However, photographs from the front line constituted only 6% of all photographs, and the proportion of single individuals was considerably lower in this zone (59% of photographs). Across all zones, most of the observed groups consisted of 2–3 individuals, although some of them could be parts of larger groups that were temporarily split. Therefore, we can only conclude that dogs rarely stayed together in large groups even if such groups existed. We did, however, document two groups comprising 7 and 11 individuals, respectively, all of which were pups (Figure S7). Fitness consequences of staying in social groups have not been extensively studied in domestic dogs, but it has been shown that larger groups tend to outcompete smaller ones in conflicts over food and space (Bonanni et al. 2011), suggesting that collective defence of resources may be one of the most important benefits of grouping (Bonanni and Cafazzo 2014). Therefore, staying in larger groups may be particularly beneficial for pups, who are not able to defend resources individually against adult dogs. The method we applied in this study was insufficient to obtain any additional information about the social groups beyond their existence and age composition, but the existence of pup‐only groups sheds light on mechanisms of group formation that could be studied elsewhere.

### 4.6 Conclusion

Dog populations living in regions differing in the magnitude of war‐inflicted environmental damage showed substantial differences in the frequencies of all morphological traits studied. These differences could not be explained solely by natural environmental variation resulting from climatic differences between the regions. Many traits showed a decrease in diversity and an increase in the frequencies of particular phenotypic variants along a gradient from relatively safe territories in the west to the front line in the east. Furthermore, variants that increased in frequency were typically those predominant in the entire population. The typical dog at the front line had a small height (20–40 cm), regular leg length, mesocephalic snout shape, straight ears, half‐curly tail, straight, middle‐length hair, and tan coat colour with a small percentage of white patches. The observed patterns are consistent with strong selection on the phenotypic traits in dogs living close to the front line. Given the short time frame considered, this selection was based on differential mortality rather than differential reproduction.

Populations from the three zones also differed in the age structure, frequency of ill or injured individuals, as well as BMI and stable isotope composition. The front‐line population showed a higher frequency of young individuals and a lower frequency of individuals with visible diseases or injuries compared with the populations from two other regions. This population also showed low BMI values, which declined sharply at the front line compared with the neighbouring areas to the west, mirroring the sharp increase in the number of war‐related violent events. Very low average value of BMI (~1.5) in the front‐line population was indicative of malnutrition. Stable isotope analysis suggested a higher proportion of plant‐based food in the diet of all Ukrainian dogs compared with dog populations from other countries or earlier historical periods. The differentiation in the isotopic signature between the front‐line population and the other Ukrainian populations was consistent with starvation and/or an increase in trophic level due to hunting and scavenging. These two are not mutually exclusive, as hunting or scavenging may be triggered by severe scarcity of other food sources. In the case of dogs that could be sampled for the stable isotope analysis, hunting or scavenging must have been limited, given the low trophic level indicated by the isotopic signature.

This study sheds light on how the war‐inflicted environmental damage affects animals. We documented significant effects on the frequencies of morphological traits consistent with mortality‐based selection at the front line, as well as effects on age and individual condition (diseases, BMI) consistent with high mortality of old and ill or injured individuals, malnutrition and a diet low in animal protein. Because of their close association with humans, including the dependence on anthropogenic food, dogs may experience the consequences of war in a more direct and immediate way than other domestic animals and wildlife. However, the conclusion that the war can be a factor of strong natural selection based on differential mortality is not limited to this particular species. This conclusion implies that the effects of wars on survival and evolutionary patterns in animal species are comparable to the effects of large‐scale natural or anthropogenic disasters. Consideration of the consequences of military conflicts from the perspective of “human's best friend” allows us to define them as intentionally created anthropogenic disasters, which undermines the credibility of narratives used to justify the forcible acquisition of land.

## Conflicts of Interest

The authors declare no conflicts of interest.

## Supporting information


**Data S1:** Supplemental experimental procedures.

## Data Availability

The datasets generated in this study are available in the Data S1.
