# Supplementary material for: Dogs of War: The Effect of War‐Inflicted Environmental Damage on Free‐Ranging Domestic Dogs
Source: Evol Appl. 2025 Dec 5;18(12):e70182. doi: 10.1111/eva.70182 (PMC12679485; doi:10.1111/eva.70182)
Supplement: Supplementary file 1 — Data S1: Supplemental experimental procedures. [file EVA-18-e70182-s001.zip › eva70182-sup-0001-Supinfo.pdf]

Dogs of war: The effect of war-inflicted environmental damage on free-ranging domestic dogs

Supplementary Materials

<u>Analysis of differentiation of phenotypic traits in dogs from three regions of Ukraine</u>

We obtained the quantitative measures of the height and weight of individuals, and based on these values we calculated the body mass index (BMI). Using Anderson-Darling normality test, we found that the weight did not have normal distribution, while for the height and BMI we could not reject the null hypothesis of normal distribution (Table S5). There were considerable differences in height, weight and BMI between dogs from the three zones, FL, DT and PST (Table S6). We found statistically significant differentiation between the three regions in the height, weight and BMI distribution for individuals studied using Kruskal-Wallis test (Table S7) and one-way analysis of variance (ANOVA) for normally distributed body measurements (Table S8). Significance of differentiation between pairs of regions was assessed using post-hoc Dunn's test (Table S9) and post-hoc Tukey test for normally distributed body measurements (Table S10).

We also measured the length of a front leg and calculated the proportion of the leg length to body height. We analysed this proportion as a continuous variable, but also used it to create two categories of leg length. If leg length was less than 50% of the body height, legs were classified as short, otherwise they were classified as regular length. The proportion of dogs with short legs in the entire population was 14%, and a declining trend was observed for this trait in the west-east gradient, from in 18% PST to 9% in FL (Table S12). We found statistically significant differences between the three zones in the distribution of the proportion of leg length to body height (Table S7).

We created categories for height, weight and BMI based on ranges of values and calculated frequencies of individuals within each category. Based on these frequencies, we calculated the Simpson's (1949) index D, and used 1-D as a measure of diversity for each trait. These diversity values are shown for each of the three regions and for the entire population (Table S11). We did not calculate the diversity indices for the leg length categories, because this trait had only two states (short vs regular length).

We assessed a range of phenotypic traits: snout shape, ears, hair length and structure, and coat colour, for dogs from the three zones, FL, DT and PST (Tables S14 and S15). The coat colour was assessed using the system developed for another study (Cimarelli and colleagues, unpublished), shown in Table S1. To test for the differentiation among the FL, DT and PST zones in the phenotypic traits assessed (snout shape, ears, hair length and structure, coat colour), we carried out permutational multivariate analysis of variance (PERMANOVA) (Anderson 2001) based on Bray–Curtis dissimilarities. We found significant differences among the three zones (Table S16). The post-hoc pairwise tests showed significant differentiation between all population pairs (Table S17). Similarity Percentage (SIMPER) analysis partitioning Bray-Curtis dissimilarity showed that the phenotypic traits with the greatest contribution to the differentiation between populations were coat colour, hair length and ear shape, and the specific states of these traits that had greatest contributions to the differentiation were tan & black and tan coat colour, middle hair length and straight ears (Table S18).

The tail shape was considered for a subset of individuals for which the other traits were assessed (Table S19). Differences in frequencies of different tail shapes among the three zones were statistically significant (PERMANOVA, $R2 = 0.025$, $F=3.88$, $P=0.006$, $N=305$; Table S20). Snout shape, tail shape, hair type and length, basic coat colour, additional colour patterns and the proportion of white patches showed a decrease in diversity, assessed using the Simpson's (1949) index, in FL compared with DT and PST (Table S11). The ear shape was the only phenotypic trait among those assessed that did not decrease in diversity in FL.

We also assessed the proportions of individuals from different age categories (pups, adults and old) as well as individuals with visible injuries and diseases among dogs from the three zones (Table S22).

Differentiation among FL, DT and PST populations for the age class distribution, assessed with PERMANOVA based on Bray–Curtis dissimilarities, was statistically significant (Table S23). However, the post-hoc pairwise tests showed significant differentiation only between DT and PST, while the differentiation between other pairs of populations was non-significant (Table S24).

Differentiation in the proportions of dog with different types of injuries/diseases among FL, DT and PST zones, assessed with PERMANOVA based on Bray–Curtis dissimilarities, was non-significant (Table S25). Significance of pairwise differentiation between zones was assessed using post-hoc Dunn's test (Table S26).

The choice of the spatial interpolation method

Spatial distribution maps were produced for the quantitative morphological traits and for the distribution of war-related violent events by carrying out an Inverse Distance Weighting (IWD) interpolation in ArcGIS Pro. IDW interpolates the values based on proximity to the sampling locations, with a user defined distance weighting parameter. Our purpose was to apply a smoothing function to the spatial distribution of our data points, which was needed because many dogs were sampled in the same place, but their ranging areas were larger. The resulting interpolation maps were validated by comparing them with individual data points. The map we created using the IDW interpolation identified the location of the frontline accurately in relation to the raw data on media reports, and was consistent with reports from the frontline, based on which we identified the frontline zone.

Stable isotope analysis

For a subset of dogs (97 individuals), we obtained the data on stable isotope composition of carbon and nitrogen in hair samples (Table S2). Lilliefors (Kolmogorov-Smirnov) normality test rejected the hypothesis of normal distribution for $\delta^{13}C$ and $\delta^{15}N$ values (D=0.31, P=2.2e-16). Kruskal-Wallis test showed significant differentiation of $\delta^{13}C$ and $\delta^{15}N$ values between dogs from the FL, DT and PST zones (Table S27). Significance of pairwise differentiation between zones was assessed using post-hoc Dunn's test (Table S28).

We assessed temporal changes in individual diet of Ukrainian dogs based on the differences between the values obtained from the two parts of the hair: the hair tip (older part) and the hair base (more recent part). In a study on captive wolves, the growth rate of body hair was estimated at 1.0 ± 0.07 mm/day in the summer and 0.63 ± 0.23 mm/day in the autumn (McLaren et al., 2015). Dogs in our study were sampled from spring to winter, so they experienced both hair growth rates. All hair collected were less than 5 cm long, and even assuming the slower growth rate, the maximum period covered was about 2.5 months, which does not cover the time before the war.

We found that the average difference between the older and the most recent part was 0.072‰ for $\delta^{13}C$ and -0.073‰ for $\delta^{15}N$. The difference in $\delta^{13}C$ was positive for 58.8% of samples and the difference in $\delta^{15}N$ was negative for 54.6% of samples. When each of the five regions was considered separately, the average change in $\delta^{15}N$ values was negative in four of them (except Lviv region), while there was no clear trend in the change in $\delta^{13}C$ values.

We also used 20 whisker samples to assess temporal changes in diet reflected in the isotopic composition along the whisker length, which ranged from 2.45 cm to 8.85 cm, with an average of 4.59 cm. Assuming the growth rate for whiskers of 0.37 – 0.49 mm/day (McLaren et al., 2015), the growth time of the whiskers studied was between 1.5 and 8 months. This did not cover the time before the war. However, for recently translocated individuals, changes in isotopic signatures along the hair/whisker length may reflect changes in diet associated with the translocation. Depending on their length and width (which determined the weight of each sample available for the analysis), the whiskers were cut into 2-6 pieces, 7.5 to 33 mm long, and each piece was analysed separately. For longer whiskers, which could be divided into three to six fragments, a growing trend was observed

for $\delta^{13}C$ values in 82% of samples and no clear trend was observed for $\delta^{15}N$ values. For whiskers which were divided into only two fragments (which constituted 45% of all whiskers), no trend was found either for $\delta^{13}C$ or $\delta^{15}N$ values.

Analysis of differentiation in group sizes

Based on photographs of stray dogs from across Ukraine, we assessed the proportion of dogs occurring alone or in social groups and the sizes of such groups. The frequency of solitary individuals decreased along the PST-DT-FL gradient from 91% to 59% of photographs, while the frequencies of pairs, trios and larger groups increased (Table S29). We found significant differences in the distribution of social group sizes (including solitary individuals) between the three regions (Tables S30-S31).

References

Anderson, M. J. (2001). A new method for non-parametric multivariate analysis of variance. Austral Ecology 26 (1): 32–46.

McLaren, A. A., Crawshaw, G. J., & Patterson, B. R. (2015). Carbon and nitrogen discrimination factors of wolves and accuracy of diet inferences using stable isotope analysis. Wildlife Society Bulletin, 39(4), 788-796.

Simpson, E. H. (1949). Measurement of diversity. Nature, 163, 688. doi:10.1038/163688a0

Table S1. Coat colour categories considered. This system of categorisation has been designed for another study (Cimarelli and colleagues, unpublished). We modified the original categorisation of white markings by removing “0” category meaning no white colour, because this category prevented us from using photographs showing incomplete individuals (e.g. when tip of the tail, which could have been white, was missing from the photograph).

**Groups of categories**	**Categories**	**Comments**
Basic coat colour	T: tan	pale cream/yellow/agouti/sable/red/tan
	B&T: black and tan	as above, with black back/saddle
	B: brown	chocolate brown, different from sable or tan
	BL: black	complete black, no tan shades
	AW: all white	all white but with pigmented skin, dark eyes and dark nose
	AL: albino*	all white but also no colour on skin, blue eyes and pink nose
	GR: grey	blue/grey colour
	M: merle*	variegated coat pattern with irregular patches of various colours (includes harlequin and tweed)
Additional coat colour pattern	BR: brindle	irregular pattern of pheomelanin and eumelanin stripes in varying shades, can be all brindle or brindle patches
	TR: ticked or roan	presence of pigmented hair where otherwise white (e.g. spaniels and cockers)
	D: Dalmatian*	typical Dalmatian pattern
	N: none	if no secondary colour is present
White markings	1	0-20% of white colour
	2	20-40% of white colour
	3	40-60% of white colour
	4	60-80% of white colour
	5	80-100% of white colour

* These traits were absent from the study population.

Table S2. Stable isotope composition of carbon and nitrogen in hair samples of Ukrainian dogs. Each hair sample was divided into two parts, which were measured separately. Mean values from these two measurements are reported.

Sample ID	location	zone	$\delta^{13}C$ mean	$\delta^{15}N$ mean
DH1	Dnipro	DT	-19.7156484	7.45211961
DH2	Dnipro	DT	-19.3160247	7.07894302
DH3	Dnipro	DT	-19.6799677	8.81068411
DH4	Dnipro	DT	-18.8919342	7.14702253
DH5	Dnipro	DT	-20.2233336	8.19998566
DH6	Dnipro	DT	-19.5719062	8.29680986
DH7	Dnipro	DT	-20.0723939	7.07529417
DH8	Dnipro	DT	-20.0315762	6.79936045
DH9	Dnipro	DT	-19.9581043	6.79533222
DH10	Dnipro	DT	-20.0825984	6.61406190
DH11	Dnipro	DT	-19.6483996	7.12161880
DH12	Dnipro	DT	-19.8029968	6.57377960
DH13	Dnipro	DT	-19.6968707	6.93078144
DH14	Dnipro	DT	-19.9131716	6.54615817
DH15	Dnipro	DT	-19.7040138	6.76864520
DH16	Dnipro	DT	-20.0249433	7.66744389
DH17	Dnipro	DT	-19.7473827	6.48465503
DH18	Dnipro	DT	-19.4727308	6.83431074
DH19	Dnipro	DT	-20.9739558	8.50531561
DH20	Dnipro	DT	-19.6611552	6.99170841
DH22	Dnipro	DT	-19.8519781	6.85776978
DH23	Dnipro	DT	-20.1132117	8.48366387
DH24	Dnipro	DT	-20.0310660	6.78072989
DH25	Dnipro	DT	-19.6881969	6.99221193
DH26	Dnipro	DT	-19.6570301	7.29881463
DH27	Dnipro	DT	-19.2574064	6.90546634
DH28	Dnipro	DT	-19.5678284	7.14702253
DH29	Dnipro	DT	-19.8390017	7.05372839
DH30	Dnipro	DT	-19.2370175	6.80158204
DH31	Dnipro	DT	-20.0535955	6.84293404
DH32	Dnipro	DT	-19.8634684	6.81418936
DH33	Dnipro	DT	-19.7982237	7.38202293
DH34	Dnipro	DT	-19.6126841	6.47278321
DH35	Dnipro	DT	-20.3507422	7.03554857
DH36	Dnipro	DT	-19.7276001	7.22998448
DH37	Dnipro	DT	-19.8698004	6.72798631
DH38	Dnipro	DT	-20.2684692	6.70222986
DH39	Dnipro	DT	-19.9759428	7.64006749
DH40	Dnipro	DT	-19.0810419	7.87572547
DI1	Donetsk	FL	-20.5118375	8.23276469
DI4	Donetsk	FL	-20.9603947	7.92413756
DI7	Donetsk	FL	-20.9710990	8.25394498
DI9	Donetsk	FL	-20.9394961	7.99322566
DI10	Donetsk	FL	-20.8961820	8.68800130
DI11	Donetsk	FL	-19.8828379	6.87117443
DI12	Donetsk	FL	-21.4050559	7.65370325
DI13	Donetsk	FL	-21.2004748	7.47733424
DI15	Donetsk	FL	-20.7534738	7.61637504
P1	Lviv	PST	-20.2249143	5.66483881

P2	Lviv	PST	-19.5362362	5.89794062
P3	Lviv	PST	-19.7031940	6.81419537
P4	Lviv	PST	-21.2457612	6.98366730
P5	Lviv	PST	-22.0713318	7.87779515
P6	Lviv	PST	-21.3720452	7.15372520
P7	Lviv	PST	-17.9384154	7.63350212
P8	Lviv	PST	-20.7807377	7.47448214
P9	Lviv	PST	-21.2114478	7.80286543
P10a	Lviv	PST	-19.2567011	6.67816392
P11	Lviv	PST	-20.0196186	7.35523150
P12	Lviv	PST	-20.1815363	7.41782771
P13	Lviv	PST	-21.0236005	8.17527532
P14	Lviv	PST	-21.5328149	7.60290312
T11a	Truskavets, Lviv	PST	-18.7486779	6.81984203
T12a	Truskavets, Lviv	PST	-19.7331638	6.75201740
T13a	Truskavets, Lviv	PST	-19.6318646	7.58600914
T14a	Truskavets, Lviv	PST	-20.4188425	6.90173399
T15a	Truskavets, Lviv	PST	-20.4025532	7.09315017
T16a	Truskavets, Lviv	PST	-22.6932393	7.19162148
T17a	Truskavets, Lviv	PST	-19.7509803	6.62340181
T18a	Truskavets, Lviv	PST	-20.3536852	7.70005500
T19a	Truskavets, Lviv	PST	-19.0505394	6.74699336
T20a	Truskavets, Lviv	PST	-19.4957363	7.02014671
T21a	Truskavets, Lviv	PST	-21.6412394	6.57652857
T22a	Truskavets, Lviv	PST	-20.6211861	6.73802431
T23a	Truskavets, Lviv	PST	-19.6539906	6.98127727
T24a	Truskavets, Lviv	PST	-21.6869817	7.52582072
T25a	Truskavets, Lviv	PST	-19.5482751	7.09735233
T27a	Truskavets, Lviv	PST	-21.1919483	7.20383859
T28	Truskavets, Lviv	PST	-19.7235372	7.47593328
T29a	Truskavets, Lviv	PST	-21.6488631	7.69791462
T30a	Truskavets, Lviv	PST	-19.8562732	8.02342947
T31a	Truskavets, Lviv	PST	-21.4384485	7.02568860
ZA1	Zaporizhia	DT	-19.0409963	6.55219278
ZA2a	Zaporizhia	DT	-18.8525223	6.21394201
ZA3a	Zaporizhia	DT	-19.1798359	6.26619210
ZA4	Zaporizhia	DT	-18.5472929	6.86750108
ZA6a	Zaporizhia	DT	-19.3605456	6.99668847
ZA8a	Zaporizhia	DT	-19.2536469	6.50985836
ZA9a	Zaporizhia	DT	-18.7400242	6.70378656
ZA10	Zaporizhia	DT	-21.8557206	8.14985543
ZA11	Zaporizhia	DT	-21.3496994	7.70723626
ZA12a	Zaporizhia	DT	-19.2839862	6.00119999
ZA13	Zaporizhia	DT	-20.3079479	6.72795531
ZA14a	Zaporizhia	DT	-20.6465985	6.59469684
ZA15a	Zaporizhia	DT	-20.2455910	6.61236044
ZA16	Zaporizhia	DT	-19.5305511	7.54360708
ZA24	Zaporizhia	DT	-19.0886877	6.98262312

Table S3. Categories of violent events included in the GLR analyses; based of Zhukov (2023)

Variable	Description
t_aad	Anti-air defence, Buk, shoulder-fired missiles (Igla, Strela, Stinger)
t_armor	Tank battle or assault
t_artillery	Shelling by field artillery, howitzer, mortar, or rockets like Grad/BM-21, Uragan/BM-27, other Multiple Launch Rocket System (MRLS)
t_firefight	Any exchange of gunfire with handguns, semi-automatic rifles, automatic rifles, machine guns, rocket-propelled grenades (RPGs)
t_ied	Improvised explosive device, roadside bomb, landmine, car bomb, explosion

Table S4. Land use categories included in the assessment of NDVI in agricultural areas extracted from OpenStreetMap, based on data from April 2022.

Variable	Description
farmland	An area of farmland used for tillage (cereals, vegetables, oil plants, flowers)
farmyard	An area of land with farm buildings like farmhouse, dwellings, farmsteads, sheds, stables, barns, equipment sheds, feed bunkers, etc. plus the open space in between them and the shrubbery/trees around them
meadow	A meadow or pasture: land primarily vegetated by grass and non-woody plants, mainly used for hay or grazing
vineyard	A piece of land where grapes are grown

Table S5. Anderson-Darling normality test for body measurements.

Variable	Statistic	p value	Normality
Height	0.50	0.21	YES
Weight	2.29	<0.001	NO
BMI	0.58	0.123	YES
Leg/height proportion	4.54	<0.001	NO

Table S6. Average height, weight and BMI is groups of dogs from Ukraine from different zones of data collection (see Figure 1).

Zone	height	weight	BMI
FL	39.769	13.577	1.483
DT	50.671	19.958	2.496
PST	37.897	17.761	2.391
ALL	45.320	19.127	2.425

Table S7. Kruskal-Wallis test for variance between zones (FL, DT, PST) for body measurements.

Variable	Kruskal-Wallis chi-squared	df	P-value
BMI	20.40	2	3.72e-05
Weight	10.19	2	0.0061
Height	31.40	2	1.52e-07
Leg/height proportion	12.67	2	0.0018

Table S8. One-way analysis of variance (ANOVA) between zones (FL, DT, PST) for normally distributed body measurements.

Variable	Df	Sum Sq	Mean Sq	F value	P(>F)
BMI	2 13.37	11.18	6.686	11.18	2.1e-05
Height	2	7712	3856	17.1	9.36e-08

Table S9. Post-hoc Dunn (1964) Kruskal-Wallis multiple comparisons between zones (FL, DT, PST) for body measurements; p-values adjusted using the Bonferroni method.

Variable	Comparison	Z	P unadj.	P adj.
BMI	DT-FL	3.93	8.60e-05	0.00026
	DT-PST	2.76	5.74e-03	0.017
	FL-PST	-2.75	5.93e-03	0.018
Height	DT-FL	2.78	5.45e-03	1.64e-02
	DT-PST	5.23	1.70e-07	5.10e-07
	FL-PST	-0.51	6.075e-01	1.00e+00
Weight	DT-FL	2.88	0.0040	0.012
	DT-PST	1.66	0.097	0.29
	FL-PST	-2.25	0.024	0.07
Leg/height proportion	DT-FL	2.53	0.011	0.035
	DT-PST	2.97	0.0029	0.0088
	FL-PST	-1.16	0.24	0.73

Table S10. Post-hoc Tukey multiple comparisons of means between zones (FL, DT, PST) for normally distributed body measurements.

Variable	Comparison	diff	lwr	upr	P adj.
BMI	DT-FL	-1.034	-1.60	-0.47	0.000063
	DT-PST	-0.24	-0.47	-0.014	0.034
	FL-PST	0.80	0.22	1.37	0.0038
Height	DT-FL	-10.21	-20.36	-0.062	0.050
	DT-PST	-10.46	-14.83	-6.095	0.0000001
	FL-PST	-0.25	-10.68	10.17	0.10

Table S11. Diversity within each phenotypic trait, measured as 1-D, where D is the Simpson's index.

Zone	height	weight	BMI	snout shape	ear shape	tail shape	hair type	hair length	basic coat colour	coat colour patterns	percentage of white patches	diseases	age
FL	0.722	0.497	0.446	0.000	0.686	0.449	0.126	0.462	0.575	0.053	0.327	0.053	0.362
DT	0.800	0.859	0.776	0.093	0.686	0.552	0.174	0.551	0.615	0.162	0.439	0.247	0.317
PST	0.806	0.836	0.837	0.089	0.680	0.598	0.196	0.642	0.624	0.056	0.390	0.203	0.203
ALL	0.824	0.856	0.813	0.083	0.687	0.557	0.175	0.587	0.617	0.127	0.417	0.218	0.297

Table S12. Frequencies of leg length types in domestic dogs from Ukraine grouped according to the zone of data collection. Leg proportions were calculated for dogs with measured height and leg length. Leg proportion was defined as leg length divided by height; if it was >0.5, the legs were considered normal, and if ≤0.5, the legs were considered short.

Zone	N	mean leg proportion	normal leg	short leg
FL	11	0.57	**0.91**	0.09
DT	107	0.66	**0.90**	0.10
PST	88	0.59	**0.82**	0.18
ALL	206	0.62	**0.86**	0.14

Table S13. GLR models showing correlations between dog BMI, the number of war-related violent events and the mean annual temperature.

Dog trait	Explanatory Variable	Coefficient	StdError	t-Statistic	Probability	Robust_SE	Robust_t	Robust_Pr	VIF
BMI	Intercept	0.001009	0.022678	0.044512	0.964489	0.022682	0.044504	0.964496	--------
BMI	SUMEVENTS	-0.464705	0.022850	-20.336797	0.000000*	0.024576	-18.908997	0.000000*	1.011283
BMI	TEMPERATURE	0.275975	0.022813	12.097211	0.000000*	0.021401	12.895640	0.000000*	1.011283

Table S14. Frequencies of phenotypic traits in domestic dogs from Ukraine grouped according to the zone of data collection. Traits showing a trend of increase from PST to FL are marked in bold. In "N" column the maximum sample size is reported. Information on snout shape was missing for one DT individual. Information on ear shape was missing for 3 FL, 13 DT and 3 PST individuals.

		snout shape			ear shape						hair type			hair length			
Zone	N	brachy-cephalic	meso-cephalic	dolicho-cephalic	straight	half-floppy	floppy	straight/ half-floppy	half-floppy/ floppy	cut	straight	half-curly	curly	bold	short	middle	long
FL	74	0.000	1.000	0.000	**0.408**	0.282	0.254	0.056	0.000	0.000	**0.932**	0.068	0.000	0.000	0.149	**0.703**	0.149
DT	474	0.044	0.951	0.004	**0.382**	0.223	0.341	0.043	0.004	0.004	**0.905**	0.082	0.013	0.002	0.228	**0.608**	0.167
PST	174	0.034	0.954	0.011	**0.351**	0.246	0.368	0.018	0.006	0.012	**0.891**	0.103	0.006	0.006	0.431	**0.356**	0.213
ALL	722	0.037	0.957	0.006	**0.377**	0.235	0.339	0.038	0.004	0.006	**0.904**	0.086	0.010	0.003	0.269	**0.557**	0.176

Table S15. Frequencies of coat colour types in domestic dogs from Ukraine grouped according to the zone of data collection. Coat colour was assessed in three categories: basic colour, colour patterns (brindle, ticked or roan) and percentage of the total coat surface covered by white colour. Traits showing a trend of increase from PST to FL are marked in bold. In "N" column the maximum sample size is reported. Information on the percentage of white patches was missing for two DT individuals. The colour categories are listed in Table S1.

		basic coat colour						colour patterns			percentage of white patches				
Zone	N	tan	black & tan	brown	black	all white	grey	brindle	ticked/roan	none	0-20	20-40	40-60	60-80	80-100
FL	74	**0.541**	0.351	0.014	0.095	0.000	0.000	0.014	0.014	0.973	0.811	0.108	0.054	0.027	0.000
DT	474	**0.517**	0.306	0.013	0.156	0.006	0.002	0.004	0.084	0.911	0.729	0.155	0.047	0.059	0.008
PST	174	**0.431**	0.414	0.000	0.138	0.011	0.006	0.000	0.029	0.971	0.770	0.109	0.040	0.052	0.029
ALL	722	**0.499**	0.337	0.010	0.145	0.007	0.003	0.004	0.064	0.932	0.747	0.139	0.046	0.054	0.013

Table S16. PERMANOVA table of results for the zone (FL, DT, PST) factor explaining variability of genetically determined features (snout shape, ears, hair length and structure, coat colour).

Factor	Df	SS	R²	F	P(>F)
Zone	2	1.013	0.01636	5.7973	0.001***
Residual	697	60.883	0.98364		
Total	699	61.895	1.00000		

Table S17. Post-hoc pairwise test for the zone (FL, DT, PST) factor explaining variability of genetically determined features (snout shape, ears, hair length and structure, coat colour). P-values adjusted with the Bonferroni method.

Comparison	Df	SS	R²	F	P(>F)
DT-FL	1	0.097	0.0073	3.87	0.01
DT-PST	1	0.18	0.011	7.0656	0.001
FL-PST	1	0.23	0.040	10.13	0.001

Table S18. Similarity Percentage (SIMPER) analysis partitioning Bray-Curtis dissimilarity - cumulative contributions of the most influential phenotypic traits between zones (FL, DT, PST).

Comparison	Trait	Contribution
DT-FL	Tan coat colour	0.10
	Straight ears	0.20
	Middle hair length	0.29
DT-PST	Middle hair length	0.096
	Tan coat colour	0.19
	Straight ears	0.27
FL-PST	Middle hair length	0.11
	Tan coat colour	0.21
	Black&tan coat colour	0.30

Table S19. Frequencies of tail shape types in domestic dogs from Ukraine grouped according to the zone of data collection. The tail shape type showing a trend of increase from PST to FL is marked in bold.

Zone	N	straight	half-curly	curly
FL	51	0.216	**0.706**	0.078
DT	257	0.342	**0.568**	0.089
PST	95	0.389	**0.484**	0.126
ALL	403	0.337	**0.566**	0.097

Table S20. Post-hoc pairwise test for the zone (FL, DT, PST) factor explaining variability of tail shape; P-values adjusted with the Bonferroni method.

Comparison	Df	SS	R²	F	P(>F)
DT-FL	1	0.050	0.0023	0.478	0.48
DT-PST	1	0.820	0.0279	7.2316	0.008 **
FL-PST	1	0.77	0.043	6.53	0.008 **

Table S21. Pairwise correlation coefficients (rho) between morphological variables analysed in this study, calculated using Spearman's rank correlation test. The calculations were carried out for all samples with the complete set of data (N=395) and for subsets of samples from each zone. “NA” refers to the traits that showed no variation in the FL zone (e.g. no dogs with additional colour patterns). The stars indicate significant correlations shown for individual pairwise comparisons, with the following significance thresholds: *P<0.05; **P<0.01; ***P<0.001; ****P<0.0001. The correlations that remain significant after the Bonferroni correction are highlighted in bold.

	Snout shape	Ear shape	Tail shape	Hair type	Hair lengh	Coat colour (basic)	Additional colour pattern
All samples							
Ear shape	-0.0764						
Tail shape	-0.0573	0.0303					
Hair type	**-0.2893******	0.0966	0.1225*				
Hair lengh	**-0.1684*****	-0.0657	0.0570	**0.4518******			
Coat colour (basic)	0.0171	0.1327**	-0.0364	0.0139	-0.0003		
Additional colour pattern	-0.0483	0.0246	0.0845	0.0494	-0.0050	-0.1025*	
White markings	0.0520	-0.0806	0.0181	-0.1567**	0.0353	-0.0787	**-0.2207******
PST zone							
Ear shape	-0.1171						
Tail shape	-0.1750	-0.0062					
Hair type	-0.2718**	0.1398	0.1602				
Hair lengh	-0.2847**	-0.1183	0.1163	**0.4964*****			
Coat colour (basic)	-0.1756	0.0384	0.0424	0.0454	-0.0263		
Additional colour pattern	-0.0227	0.0652	0.0088	0.0826	-0.0086	0.0836	
White markings	0.0440	-0.3043**	-0.0138	-0.2494*	0.0542	-0.1148	-0.2437*
DT zone							
Ear shape	-0.0707						
Tail shape	-0.0238	0.0728					
Hair type	**-0.2974*****	0.0903	0.1266				
Hair lengh	-0.1258*	-0.0113	0.0259	**0.4672*****			
Coat colour (basic)	0.0808	0.1806**	-0.0696	0.0037	-0.0068		

Additional colour pattern	-0.0687	0.0224	0.1083	0.0528	0.0058	-0.1607*	
White markings	0.0743	-0.0117	0.0071	-0.1159	0.0179	-0.0642	**-0.2248*****
FL zone							
Ear shape	NA						
Tail shape	NA	-0.0896					
Hair type	NA	-0.1427	0.0442				
Hair lengh	NA	-0.1313	0.0839	0.2972*			
Coat colour (basic)	NA	0.0251	0.0093	-0.1263	0.1526		
Additional colour pattern	NA	NA	NA	NA	NA	NA	
White markings	NA	0.1091	0.1796	-0.2317	-0.0075	-0.0044	NA

Table S22. Frequencies of different types of diseases/injuries and frequencies of different age classes in domestic dogs from Ukraine grouped according to the zone of data collection. In "N" column the maximum sample size is reported. Information on the age was missing for one DT individual.

		diseases/injuries						age		
Zone	N	eye	skin	legs	wounds	other	all	young	adult	old
FL	74	0.000	0.000	0.014	0.000	0.014	0.027	0.122	0.784	0.095
DT	474	0.040	0.006	0.049	0.017	0.023	0.135	0.078	0.816	0.106
PST	174	0.011	0.006	0.017	0.034	0.040	0.109	0.000	0.885	0.115
ALL	722	0.029	0.006	0.037	0.019	0.026	0.118	0.064	0.829	0.107

Table S23. PERMANOVA table of results for the zone (FL, DT, PST) factor explaining variability of proportions of age classes.

Factor	Df	SS	R^2	F	P(>F)
Zone	2	0.89	0.0084	2.97	0.029 *
Residual	697	104.79	0.99		
Total	699	105.69	1.0		

Table S24. Post-hoc Dunn's test for the zone (FL, DT, PST) factor explaining variability of proportions of age classes; P-values adjusted with the Bonferroni method.

Comparison	Df	SS	R^2	F	P(>F)
DT-FL	1	0.016	0.00028	0.15	0.71
DT-PST	1	0.42	0.0065	4.079	0.037 *
FL-PST	1	0.28	0.012	3.019	0.084

Table S25. PERMANOVA table of results for the zone (FL, DT, PST) factor explaining variability of diseases/injuries occurrence.

Factor	Df	SS	R^2	F	P(>F)
Zone	2	0.00097	0.0033	1.1665	0.38
Residual	667	0.290519	0.99666		
Total	699	0.291492	1.00000		

Table S26. Post-hoc Dunn's test for the zone (FL, DT, PST) factor explaining variability of diseases/injuries occurrence; P-values adjusted with the Bonferroni method.

Comparison	Df	SS	R^2	F	P(>F)
DT-FL	1	0.37	0.010	5.30	0.031
DT-PST	1	0.244	0.018	4.35	0.042
FL-PST	1	0.010	0.0002	0.13	0.68

Table S27. Kruskal-Wallis test results for the zone (FL, DT, PST) factor explaining the variability of isotope composition variance.

Variable	Kruskal-Wallis chi-squared	df	P-value
Carbon	18.52	2	9.53e-05
Nitrogen	12.73	2	0.0017

Table S28. Post-hoc Dunn (1964) Kruskal-Wallis multiple comparison between zones (FL, DT, PST) for isotopes composition; p-values adjusted with the Bonferroni method.

Variable	Comparison	Z	P unadj.	P adj.
Carbon	DT-FL	3.66	0.00027	0.00077
	DT-PST	3.064	0.0022	0.0066
	FL-PST	-1.72	0.085	0.25
Nitrogen	DT-FL	-3.53	0.00042	0.0012
	DT-PST	-1.34	0.18	0.54
	FL-PST	2.61	0.0091	0.027

Table S29. Group size estimated as the number of individuals jointly occurring on photographs. The table shows proportions of photographs showing solitary individuals as well as those showing pairs, trios and larger groups. The maximum group size reported does not include females with pups.

Zone	N photos	N dogs	solitary	pair	group of 3	larger group	female with pups	maximum group size
FL	22	35	0.591	0.227	0.091	0.091	0.000	6
DT	202	281	0.866	0.040	0.029	0.040	0.025	11
PST	150	171	0.907	0.060	0.027	0.000	0.006	3
ALL	374	489	0.869	0.059	0.027	0.029	0.016	11

Table S30. PERMANOVA table of results for the zone (FL, DT, PST) factor explaining variability of dog group size.

Factor	Df	SS	R^2	F	P(>F)
Zone	2	0.1889	0.02011	3.8789	0.021
Residual	378	9.2046	0.97989		
Total	380	9.3935	1.00000		

Table S31. Post-hoc pairwise test for the zone (FL, DT, PST) factor explaining variability of dog groups size; P-values adjusted with the Bonferroni method.

Comparison	Df	SS	R^2	F	P(>F)
DT-FL	1	0.47	0.021	4.85	0.03 *
DT-PST	1	0.071	0.0027	0.92	0.294
FL-PST	1	0.65	0.041	7.75	0.005 **

Supplementary figures

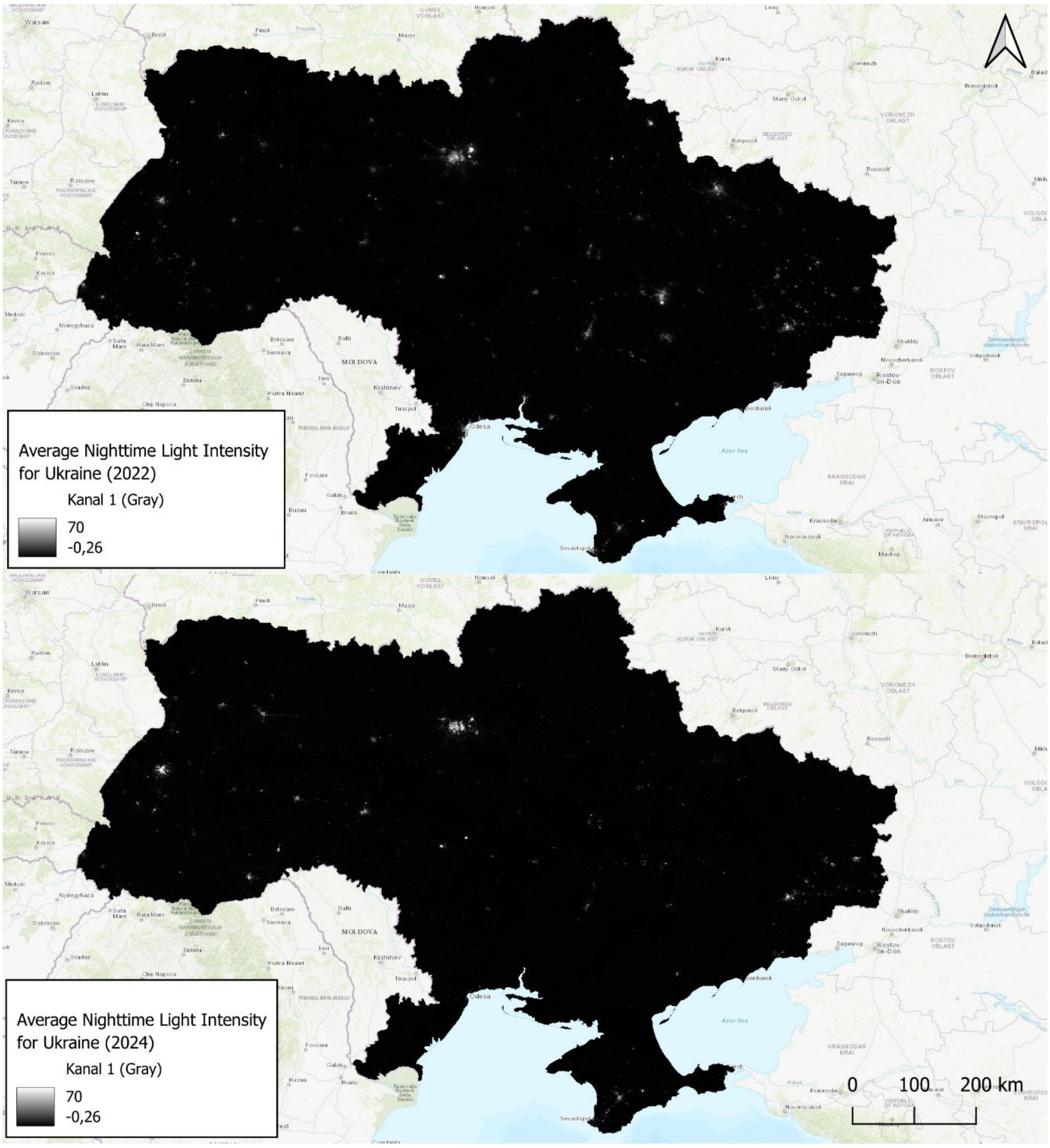

Figure S1. Nighttime Light Intensity (luminosity) in Ukraine before the war (January 2022) and at the end of our data collection (January 2024). The monthly cloud-free DNB composites with stray light correction were used to ensure the most accurate observations. The data were obtained from VIIRS (Visible and Infrared Imaging Suite) on the JPSS (Joint Polar-orbiting Satellite System) satellites. For this study, we utilized VIIRS-derived nighttime light data to analyze changes in luminosity across Ukraine, revealing trends in urbanization levels and population movement during the conflict.

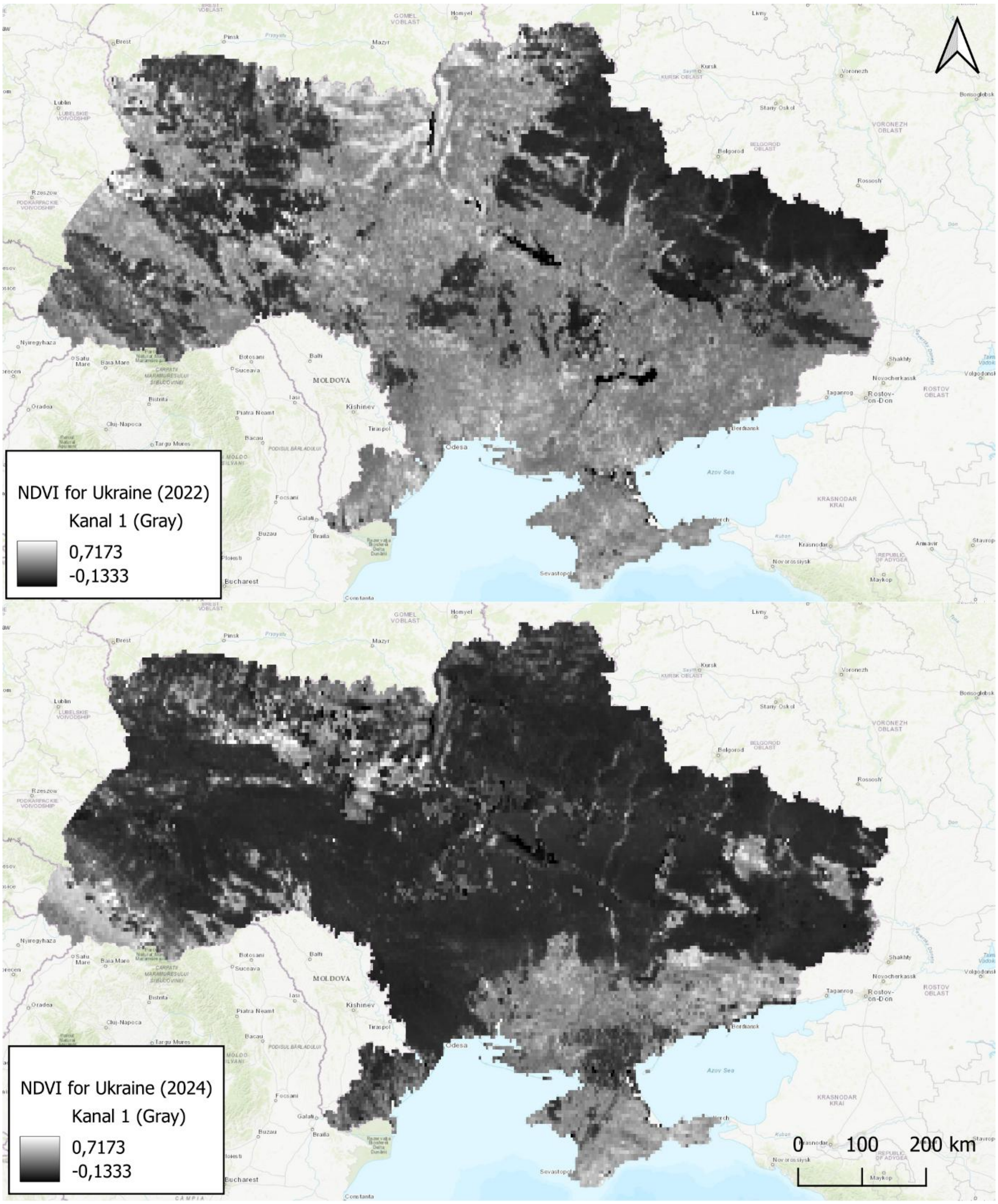

Figure S2. Normalized Difference Vegetation Index (NDVI) in Ukraine before the war (January 2022, mean NDVI= 0.26) and at the end of our data collection (January 2024, mean NDVI= 0.12). The data were obstained from MODIS database from NASA Earth Observation Data. NDVI quantifies vegetation health by analyzing the difference between near-infrared (NIR) and red light reflectance. Healthy vegetation reflects NIR and absorbs red light, with NDVI values ranging from -1 to +1. Higher values indicate dense vegetation, while lower values suggest sparse vegetation, bare soil, or water.

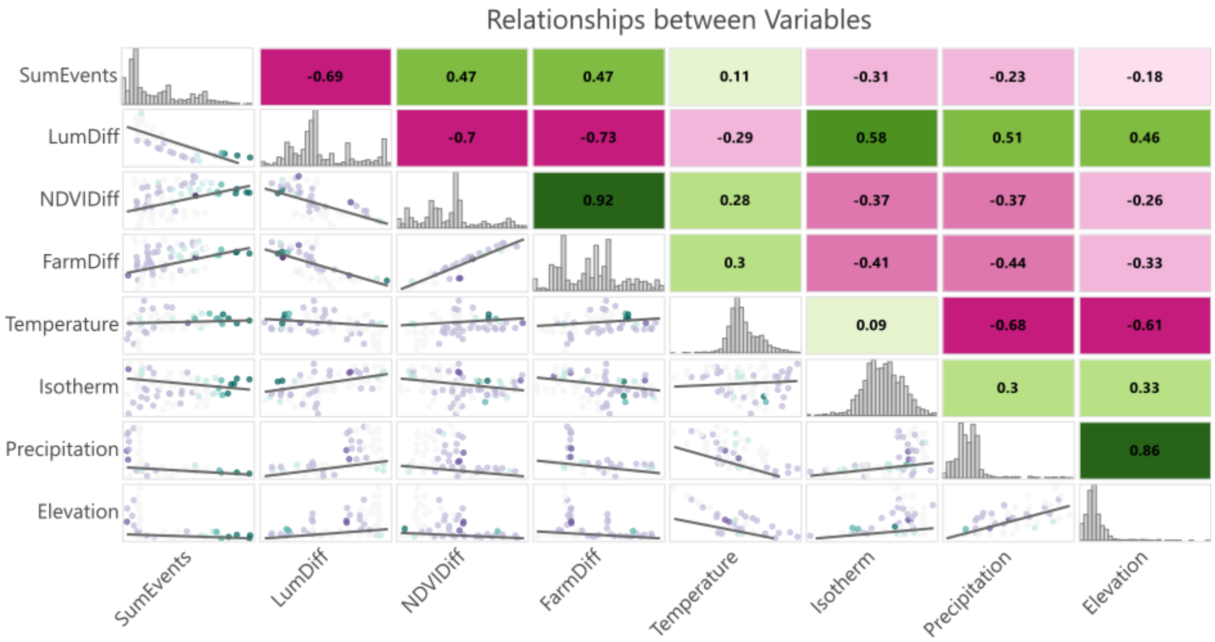

Figure S3. Correlation between the body mass index (BMI) of dogs assessed in our study and the potential explanatory variables, with the data collected within 50 km radius of the dog sampling points using ArcGIS: SumEvents - the number of violent events from the VIINA 2.0 database, LumDiff – difference in luminosity between January 2022 and January 2024 (see Figure S1), NDVIDiff – difference in NDVI between January 2022 and January 2024 (see Figure S2), FarmDiff – difference in NDVI between January 2022 and January 2024 calculated for agricultural areas only (fields, meadows, orchards), Temperature – mean annual temperature, Isotherm – isothermality, Precipitatation - mean annual precipitation, Elevation – elevation, obtained from the WorldClim database. The top right part of the matrix shows Pearson's correlation coefficient, the bottom left part shows the linear regression plots, and the diagonal shows the distributions of the traits considered.

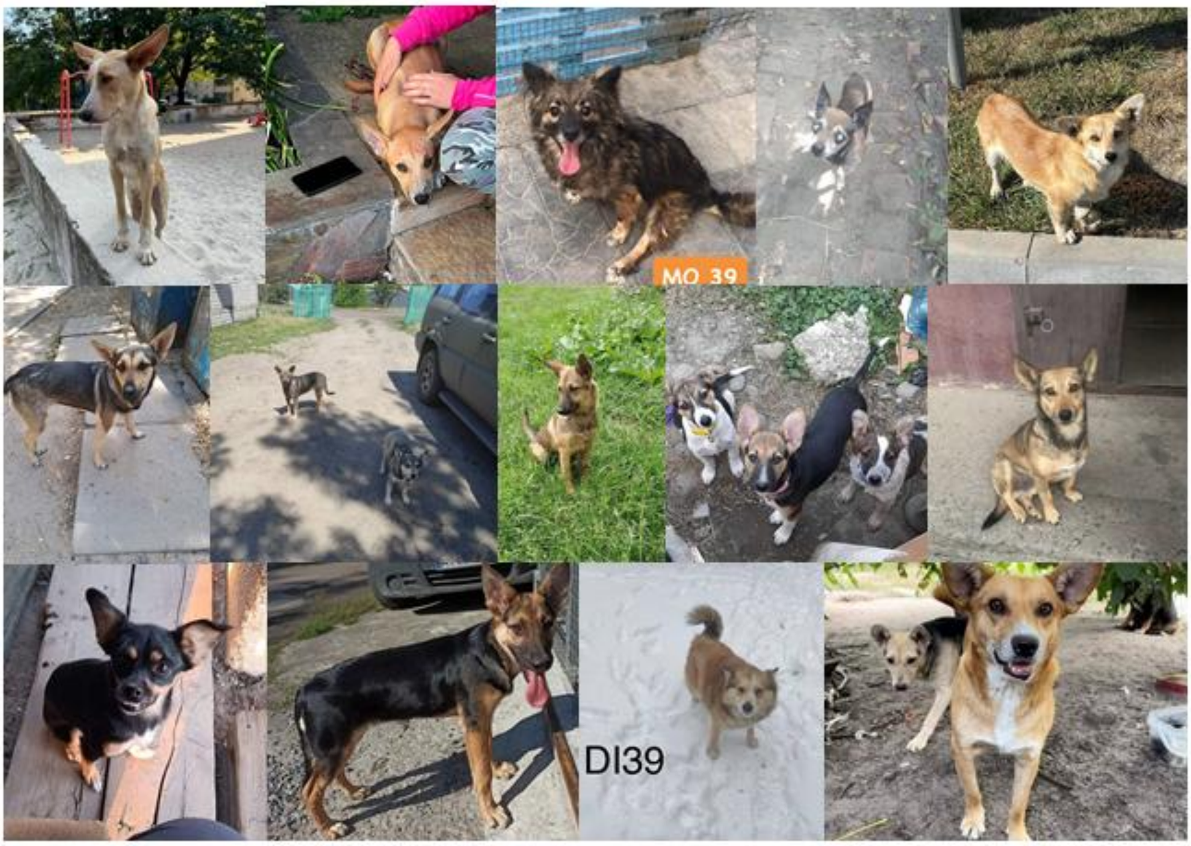

Figure S4. Example photographs of Ukrainian dogs with typical phenotypic traits.

A

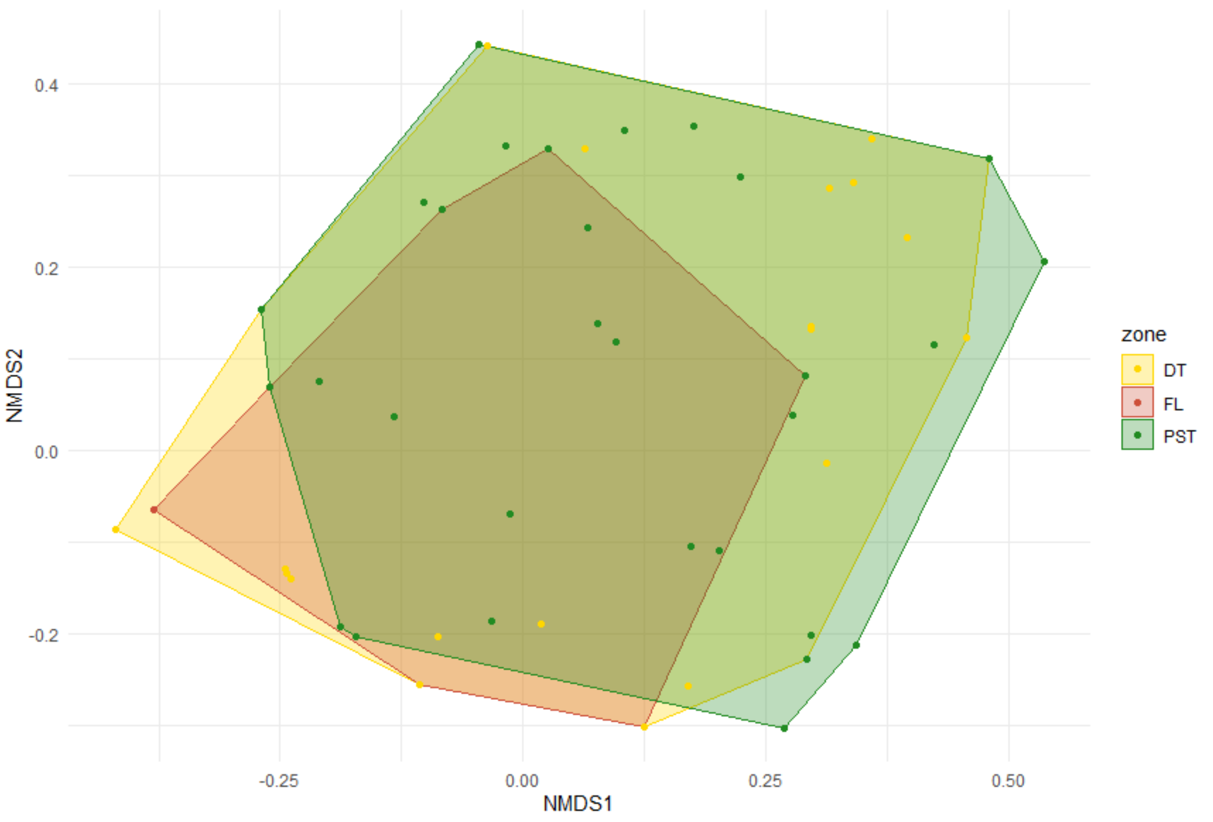

0.4
0.2
0.0
-0.2
NMDS2
-0.25
0.00
0.25
0.50
NMDS1
zone
DT
FL
PST

B

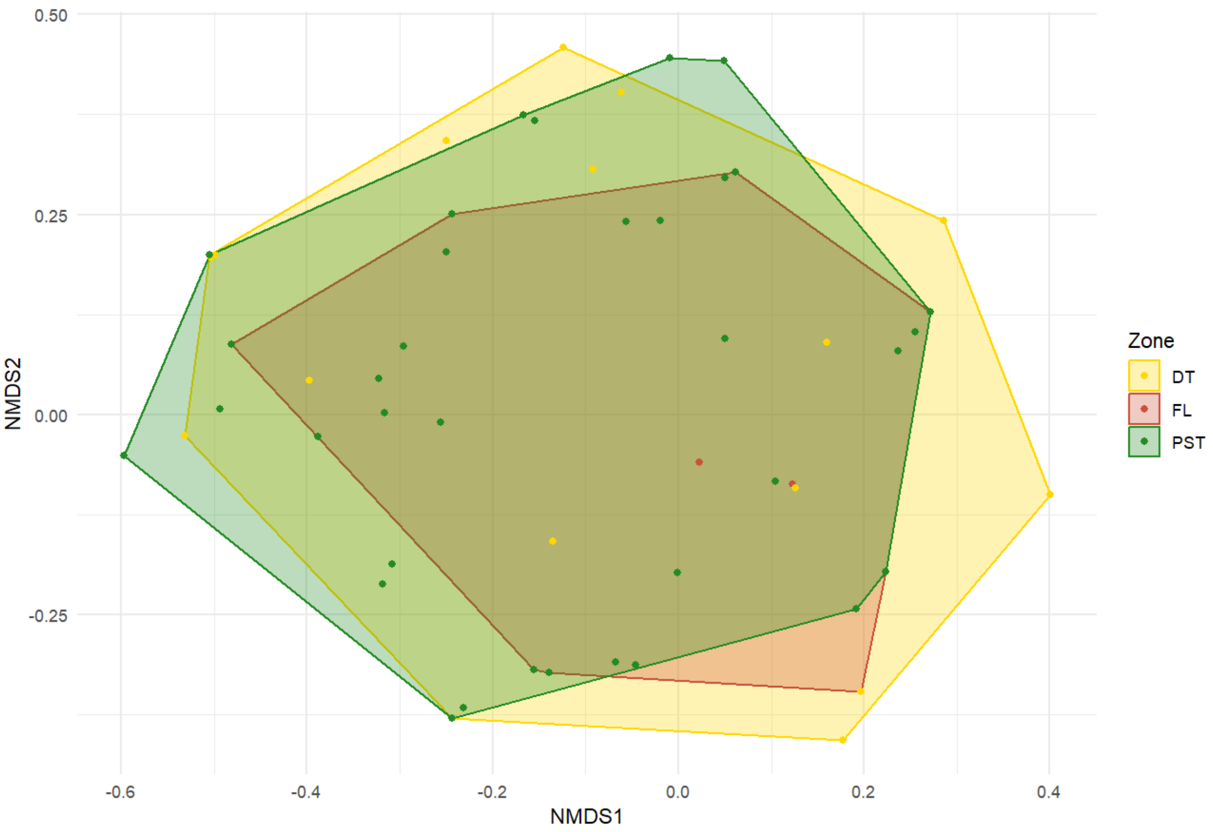

0.50
0.25
0.00
-0.25
NMDS2
-0.6
-0.4
-0.2
0.0
0.2
0.4
NMDS1
Zone
DT
FL
PST

C

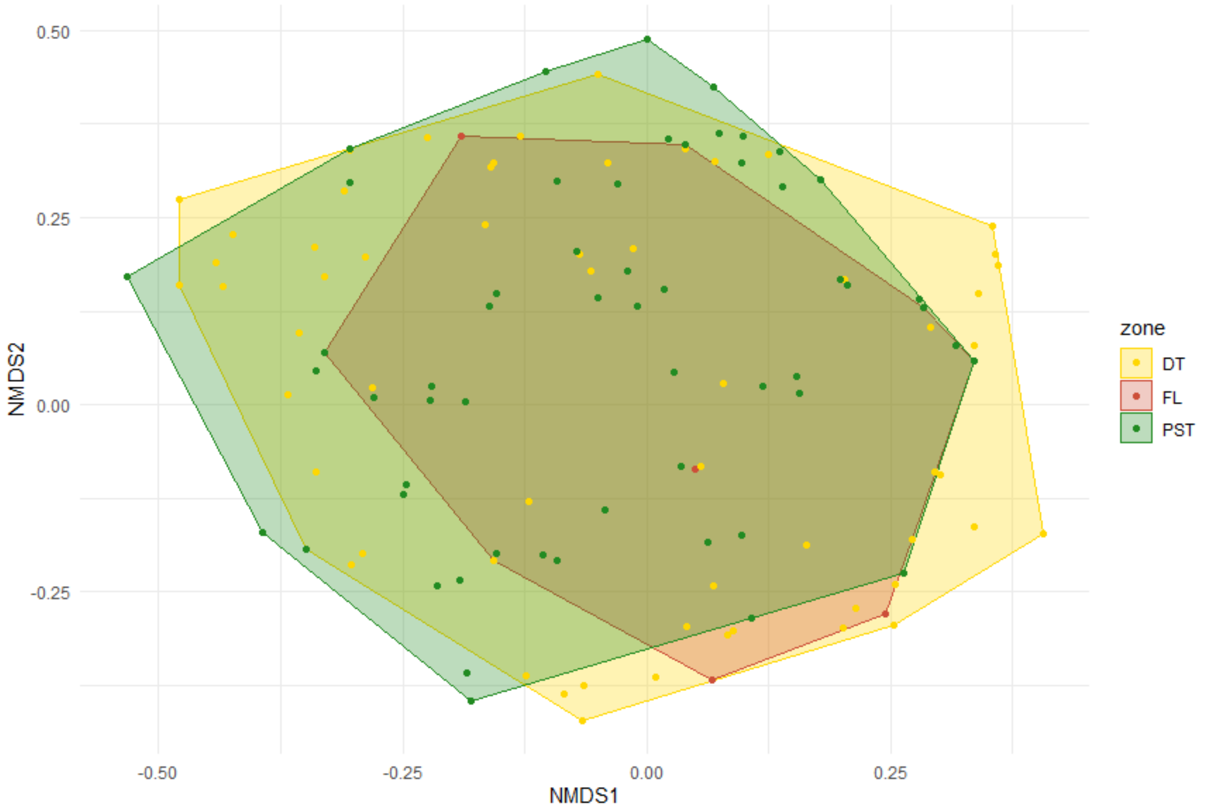

Figure S5. Nonmetric multidimensional scaling (nMDS) ordination of sampling units based on Bray–Curtis dissimilarities of the selected phenotypic traits for dogs from Ukraine (N = 700). Symbols show the prior classification of sampling sites based on the intensity of war activities: DT – dangerous territories, PST – potentially safe territories, FL – front line. Selected traits: (A) hair length, proportion of white patches, and basic coat colour – best-fitting model; stress value = 0.09; (B) hair length, ear shape and basic coat colour; stress value = 0.13; (C) ear shape, hair length, proportion of white patches, and basic coat colour; stress value = 0.14.

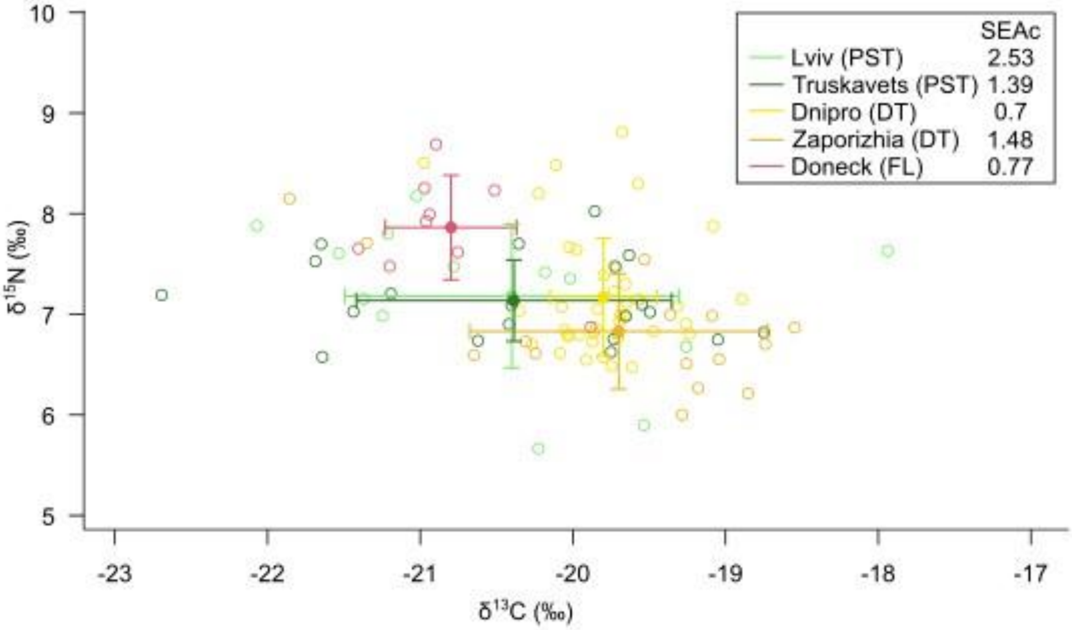

Figure S6. Stable isotope signatures of Ukrainian dogs from five location. The locations represent the three zones: PST - Potentially Safe Territories, DT - Dangerous Territories, FL - Front Line. Circles denote individual data points. Filled in circles denote averages and lines standard deviations for the locations.

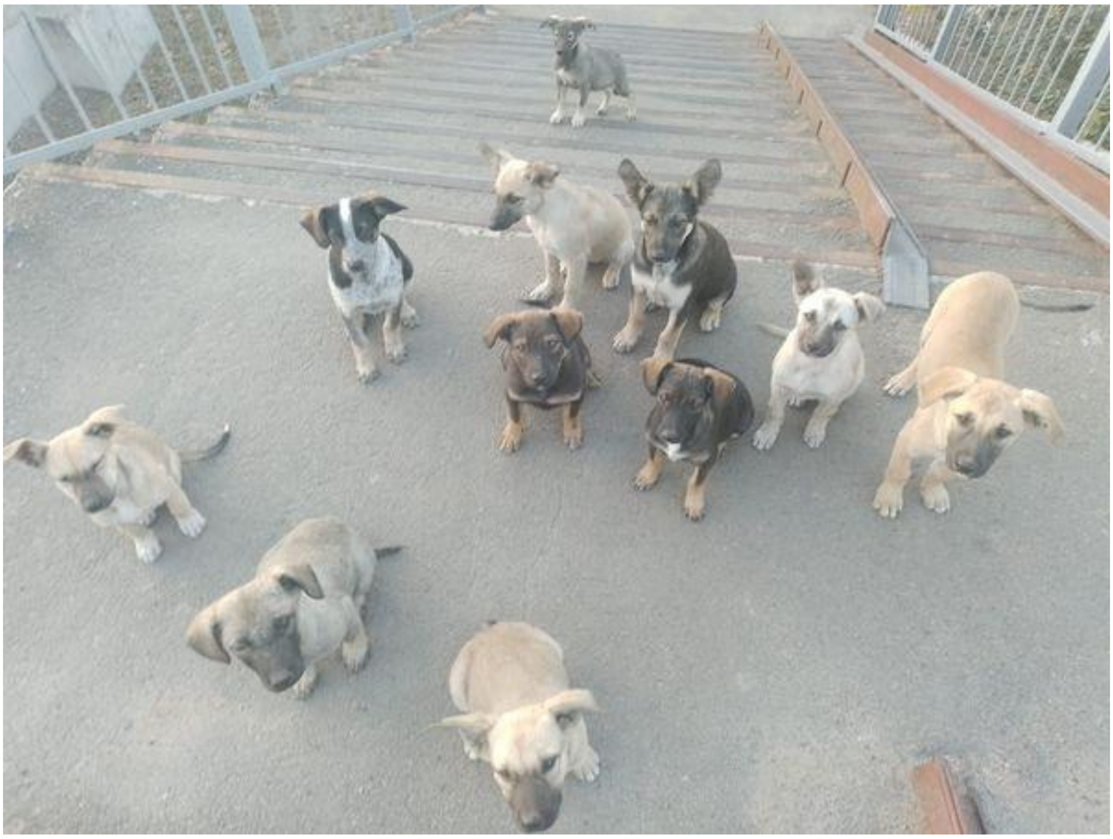

Figure S7. A group of young stray dogs consisting of pups only.
