# Supplementary figures and images for: Dogs of War: The Effect of War‐Inflicted Environmental Damage on Free‐Ranging Domestic Dogs
Source: Evol Appl. 2025 Dec 5;18(12):e70182. doi: 10.1111/eva.70182 (PMC12679485; doi:10.1111/eva.70182)

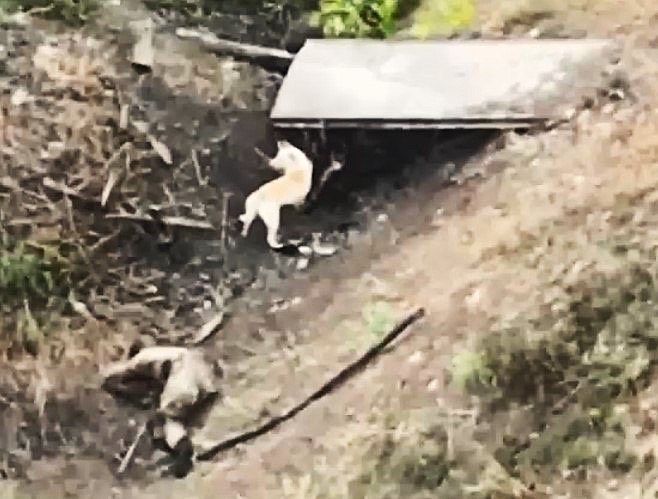

Supplement: Supplementary file 1 — Data S1: Supplemental experimental procedures. [file EVA-18-e70182-s001.zip › IMG_6585.jpeg]

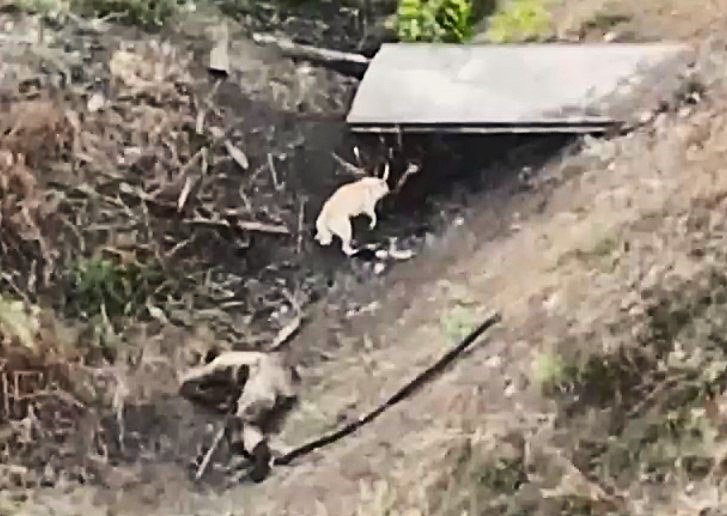

Supplement: Supplementary file 1 — Data S1: Supplemental experimental procedures. [file EVA-18-e70182-s001.zip › IMG_6586.jpeg]
